# Wulingsan alleviates cisplatin-induced acute kidney injury and inhibits renal tubular epithelial cell apoptosis in association with the CaSR/CaMKKβ/AMPK pathway

**DOI:** 10.3389/fphar.2026.1824226

**Published:** 2026-06-26

**Authors:** Xiuye Huang, Yuxian Li, Yongan Liao, Xiaotong Lin, Xipan Zhao, Xiaojie Li, Shengliang Yuan, Jiuyao Zhou

**Affiliations:** 1 School of Pharmaceutical Sciences, Guangzhou University of Chinese Medicine, Guangzhou, Guangdong, China; 2 Affiliated Gaozhou People’s Hospital, Guangdong Medical University, Gaozhou, Guangdong, China

**Keywords:** acute kidney injury, apoptosis, Ca^2+^-sensing receptor, CaSR/CaMKKβ/AMPK pathway, Wulingsan

## Abstract

**Introduction:**

The calcium-sensing receptor (CaSR), a G protein-coupled receptor (GPCR), plays a critical role in kidney injury by promoting inflammation and apoptosis when activated. Whether Wulingsan (WLS), a traditional Chinese herbal formula used for kidney disorders, exerts its protective effects against acute kidney injury (AKI) via modulation of the CaSR pathway remains unclear.

**Methods:**

WLS’s chemical profile was characterized by ultra-high performance liquid chromatography coupled with Q-Exactive mass spectrometry (UHPLC-QE-MS). C57BL/6J mice were divided into Control, cisplatin (CP)-induced AKI, WLS (2.34/4.68/9.36 g/kg), and CaSR inhibitor NPS2143 (4.5 mg/kg) groups. Mice were pretreated with WLS/NPS2143 before CP injection (20 mg/kg, i. p.). Renal function, histopathology, inflammation, oxidative stress, and apoptosis were evaluated. Cisplatin-induced mouse renal tubular epithelial cells (mRTECs) were treated with WLS-containing serum (5%–10%) for *in vitro* validation. Molecular docking predicted the binding affinity of WLS components to CaSR.

**Results:**

UHPLC-QE-MS identified 15 major WLS components, with 13 binding to CaSR (Polyporusterone E: binding energy of −10.4 kcal/mol). WLS pretreatment significantly improved renal function (reduced serum creatinine (SCr)/blood urea nitrogen (BUN)), attenuated tubular injury, suppressed inflammation (downregulated monocyte chemoattractant protein-1 (MCP-1)/tumor necrosis factor-α (TNF-α)/interleukin-1β (IL-1β)), alleviated oxidative stress (restored glutathione (GSH)/superoxide dismutase (SOD), reduced malondialdehyde (MDA)), and inhibited apoptosis (decreased terminal deoxynucleotidyl transferase dUTP nick end labeling (TUNEL)-positive cells, modulated Bcl-2-associated X protein (Bax)/B-cell lymphoma-2 (Bcl-2) ratio, downregulated cleaved Caspase 3). Mechanistically, WLS inhibited renal CaSR/calmodulin-dependent protein kinase β (CaMKKβ)/adenosine 5′-monophosphate (AMP)-activated protein kinase (AMPK) pathway activation (reduced CaSR/CaMKKβ/p-AMPK expression). *In vitro*, WLS-containing serum reduced cisplatin-induced reactive oxygen species (ROS) production, intracellular Ca^2+^ overload, and apoptosis in mRTECs, effects reversed by the CaSR agonist cinacalcet.

**Conclusion:**

This study demonstrates that WLS protects against CP-induced kidney injury and inhibits apoptosis in renal tubular epithelial cells in association with the CaSR/CaMKKβ/AMPK pathway.

## Introduction

Acute kidney injury (AKI) is characterized by a sudden decline in kidney function, with primary risk factors including severe illnesses, sepsis, hypovolaemia, acute infections, and use of nephrotoxic drugs (Kellum et al., 2021). Cisplatin, a widely used anticancer agent for solid tumors since the 1970s, causes AKI in approximately 30% of patients through mechanisms involving inflammation, oxidative stress, and apoptosis (Tang et al., 2023; Wang et al., 2023). Current therapeutic approaches for cisplatin-induced nephrotoxicity include hydration, magnesium supplementation, and mannitol-induced forced diuresis, but these remain largely supportive and often insufficient (Thompson and Joy, 2024). Therefore, new therapeutic agents are urgently needed.

Apoptosis is a key contributor to cisplatin-induced AKI (Li J. Y. et al., 2024; Wu et al., 2023). Mechanistically, cisplatin exposure triggers Ca^2+^ release from the endoplasmic reticulum into the cytosol and mitochondria, activating apoptosis signaling (Li et al., 2026). The kidney reabsorbs approximately 98% of filtered calcium, a process directly regulated by the Ca^2+^-sensing receptor (CaSR), a G protein-coupled receptor (GPCR) that orchestrates ion transport, water balance, and cellular signaling along the nephron (Gutierrez-Gallardo et al., 2026). Notably, CaSR activation has been implicated in various kidney diseases, including crystal-induced renal injury (Li et al., 2021), calcium oxalate stone formation via the protein kinase A (PKA)-forkhead box O4 (FOXO4) signaling axis (Gan et al., 2026), and calcium signaling dysregulation in diabetic nephropathy and ischemic injury (Tu et al., 2023). These evidences underscore the significant renal physiological role of CaSR and its critical involvement in the pathophysiology of various renal disorders. However, whether CaSR plays a role in cisplatin-induced AKI remains unknown.

Wulingsan (WLS), a classical Chinese herbal formula composed of five herbs: Alismatis Rhizoma. (Zexie, *Alisma orientale (Sam.)* Juzep.), Poria (Fuling, *Poriacocos* (Schw.) Wolf), Polyporus (Zhuling, *Polyporus umbellatus* (Pers.) Fries), Atractylodis Macrocephalae Rhizoma (Baizhu*, Atractylodes macrocephala* Koidz.), and Cinnamomi Cortex (Rougui, *Cinnamomum cassia* Presl.), has been widely used to treat kidney diseases including nephrotic syndrome, diabetic nephropathy, and urinary stone disease (Kim et al., 2022). Previous studies have shown that WLS regulates aquaporin expression, reduces water reabsorption, and exhibits anti-apoptotic effects in renal cells (Ahn et al., 2024; Lee et al., 2024). Our recent studies have demonstrated that WLS exerts multiple protective effects in kidney diseases: it ameliorates acute kidney injury by attenuating cellular senescence (Liu et al., 2025), and ameliorates chronic kidney disease renal fibrosis (Zeng et al., 2026). However, whether WLS inhibits apoptosis in cisplatin-induced AKI through modulation of CaSR remains unknown. To address this question, we screened 11 GPCRs reported to be expressed in kidney tissues using a cisplatin-induced AKI mouse model. CaSR emerged as the most significantly regulated GPCR by WLS and was therefore selected for further mechanistic investigation.

Hence, this study aimed to evaluate the effects of WLS on cisplatin-induced AKI and explore its underlying mechanisms. We characterized the chemical profile of WLS using UHPLC-QE-MS, investigated its protective effects *in vivo* and *in vitro*, and focused on CaSR to elucidate its involvement in the anti-apoptotic effect of WLS. To our knowledge, this is the first study to link WLS to CaSR in cisplatin-induced AKI, providing a novel mechanistic insight into its nephroprotective action.

## Materials and methods

### Reagents

The five crude herbs of WLS (Zexie, Fuling, Zhuling, Baizhu, and Rougui) were sourced from Kangmei Pharmaceutical Co., Ltd (Lot. 230404351, 230601711, 230404501, 230504211 and 230160691, respectively). Cisplatin was purchased from Aladdin (CAS No. 15663-27-1, Cat# D109812-250mg, specified as Pt content 65%, corresponding to >99% chemical purity). NPS2143 was purchased from Selleck Chemicals (purity 99.47%, S2633). Cinacalcet was purchased from MedChemExpress (purity 99.97%, HY-70037). The antibody against cleaved caspase 3 (9661s), caspase 3 (9662s), and phospho-adenosine 5′-monophosphate (AMP)-activated protein kinase α (phospho-AMPKα, 2535s) were purchased from Cell Signaling Technology (Danvers, MA, USA). The antibody against B-cell lymphoma-2 (Bcl-2, T40056) was purchased from Abmart (Shanghai, China). The antibody against Bcl-2-associated X protein (Bax, A00183) was purchased from Boster Biological Technology (Pleasanton, CA, USA). The antibodies against CaSR (19125-1-AP) and calmodulin-dependent protein kinase β (CaMKKβ, 11549-1-AP) were purchased from Proteintech Group (Wuhan, China). Anti-rabbit IgG (133650), anti-mouse IgG (115-035-003), goat-anti mouse Alexa Fluor 488 (AF488, 4408S), and goat-anti rabbit Alexa Fluor 555 (AF555) (4413S) were purchased from Jackson ImmunoResearch (West Grove, PA, USA). Antibodies against β-actin (AC026) and aquaporin-1 (AQP1, A4195) were purchased from ABclonal (Wuhan, China). The ultra-performance liquid chromatography (UPLC)-grade methanol and acetonitrile reagents were of mass spectrometry grade (Thermo Fisher Scientific Co., Ltd. USA) and the other reagents used were of analytical grade.

### Preparation of WLS aqueous extract

The five raw herbs (Zexie, Fuling, Zhuling, Baizhu, and Rougui) were weighed at a ratio of 3:5:3:2:3, ground to a fine powder and sieved. After soaking in pure water for 30 min, the powder was boiled for 30 min using a condensation reflux device. The final aqueous extract was concentrated to 1 g/mL (crude herb equivalent), aliquoted, and stored at-80 °C until use. To ensure consistency, a single batch of WLS extract was prepared. The extract was aliquoted into daily doses, stored at −80 °C, and used within 1 month. Each aliquot was thawed immediately before oral administration and was not refrozen.

### UHPLC-QE-MS analysis

The WLS aqueous extract was centrifuged for 15 min at 4 °C and 12,000 rpm. Next, 300 μL of the clear supernatant was collected and then mixed with a 4:1 mixture of methanol and water. The mixture was vortexed vigorously for 30 s, followed by ultrasonic treatment in ice water for 5 min. After centrifugation, the supernatant was passed through a 0.22 μm microporous filter and stored at −80 °C until analysis. The WLS sample was analyzed by UHPLC-QE-MS using a Thermo Fisher Scientific Vanquish UHPLC system. Chromatographic separation was performed using a Waters UPLC BEH C_18_ column (2.1 × 100 mm, 1.8 μm particle size) with 5 μL injection volume. The mobile phase consisted of two components: (A) a 0.1% formic acid solution in water, and (B) acetonitrile with 0.1% formic acid added, delivered at 0.3 mL/min. The elution gradient followed a multi-step linear protocol with the following profile: 0–1 min, 0% B; 1–2 min, 0%–30% B; 2–12 min, 30%–50% B; 12–21 min, 50%–100% B; 21–26 min, 100% B; 26–26.1 min, 100%–0% B; 26.1–30 min, 0% B. Data for MS and MS/MS were obtained via an Orbitrap Exploris 120 mass spectrometer, utilizing Xcalibur software. A Q Exactive Orbitrap operated in positive and negative electrospray modes acquired data under the following settings: sheath gas 35 arb, auxiliary gas 15 arb, capillary temperature 500 °C, spray voltage +5.5 kV (ESI^+^) or −4.5 kV (ESI^−^). Full-scan and MS/MS resolutions were 60 000 and 15 000 (FWHM), respectively. Stepped normalized collision energies were 16, 32, and 48 eV. Raw files were processed with Analyst TF 1.7 and PeakView 2.0.

### Animals

Thirty-six healthy male C57BL/6J mice (20 ± 2 g) were obtained from the Guangdong Medical Experimental Animal Center (certificate number: SYXK (Guangdong) 2019-0202). All animal experiments were strictly conducted in accordance with the approval of the Animal Ethics Committee of the Guangzhou University of Chinese Medicine (Approval Number: ZYD-2023-169) from 8 August 2023 to 1 September 2023. Animals were kept in a sterile environment at 25 °C ± 2 °C humidity (55%–65%), with a regulated light/dark cycle and unrestricted access to food and water.

After a week of acclimatization, mice were randomly assigned to six groups using a random number generator (*n* = 6): the control group, CP group, WLS low dose group (WLS-L, 2.34 g/kg), WLS medium dose group (WLS-M, 4.68 g/kg), WLS high dose group (WLS-H, 9.36 g/kg), and NPS2143 group (4.5 mg/kg). The WLS doses were calculated based on the Chinese Pharmacopoeia’s recommended human dosage (12–18 g/day). Using an average adult body weight of 70 kg, the human equivalent dose is 0.2571 g/kg/day. According to the body surface area-based dose conversion method for experimental animals, the conversion factor for mice (human to mouse) is 9.1, giving a mouse equivalent dose of 2.34 g/kg/day. This dose was used as the low dose, scaled at a 1:2:4 ratio for medium (4.68 g/kg) and high (9.36 g/kg) doses. In the WLS group, all mice were orally pre-administered the corresponding dose of WLS aqueous extract once daily for 2 weeks, as WLS is a multi-herb formula that requires accumulation time to achieve stable bioactivity, consistent with our previous study (Liu et al., 2025). In the NPS2143 group, mice were injected intraperitoneally with NPS2143 (4.5 mg/kg) (Xu et al., 2023) once daily beginning 2 days before cisplatin injection. This 2-day pretreatment was chosen to ensure that CaSR inhibition reached steady state before cisplatin challenge, given that the experiment was terminated 2 days after cisplatin injection. Subsequently, the AKI model was established by cisplatin injection (20 mg/kg, i. p.) in all mice except the control group (Liu et al., 2022) After cisplatin-induced modeling, the intervention was continued for 2 days by administering WLS or NPS2143 using the same way route and dose as before. Control and model group mice received daily distilled water by gavage, with the volume adjusted by body weight.

At the end of the study, all mice were euthanized under anesthesia by intraperitoneal injection of 1% sodium pentobarbital (50 mg/kg) to collect blood and kidney tissue in accordance with the protocol approved by the Experimental Animal Ethics Committee. The diagram of the animal experiment is shown in Figure 2A.

### Serum biochemical indexes analysis

Mouse blood was obtained from the retroorbital venous plexus and centrifuged (15 min, 4 °C) to isolate serum for subsequent assays. The resulting serum was carefully collected and then analyzed for serum creatinine (SCr) and blood urea nitrogen (BUN) levels, in accordance with the protocol provided by the Nanjing Jiancheng Bioengineering Institute.

### Histological analysis

Following fixation in 4% paraformaldehyde, kidney tissues were dehydrated, embedded in paraffin, and sectioned into 4 μm slices. Next, the sections were mounted onto slides, treated with an ethanol gradient to remove the paraffin and subsequently stained using hematoxylin-eosin (H&E). Finally, kidney tissues were examined and photographed using an optical microscope (Olympus BX53, Japan). Tubular injury was scored blindly by two independent investigators. For each kidney section, ten non-overlapping fields at 400× magnification were examined. The degree of renal cortical tubular injury was graded into 5 grades (normal, <1/4 injury, 1/4–1/2 injury, 1/2–3/4 injury, and >3/4 injury), which were scored from 0 to 4.

### Immunohistochemistry

After drying in an oven at 60 °C for 1 h, sections were dewaxed using graded benzene and ethanol solutions. The sections were incubated with 3% H_2_O_2_ at room temperature for 10 min before being subjected to boiling heated with sodium citrate to boiling for an additional 5 min. After washing with PBS, the sections were blocked with 5% bovine serum albumin (BSA) at 37 °C for 15 min and incubated with anti-CaSR and anti-CaMKKβ antibodies at 4 °C overnight. After another PBS wash, the sections were incubated with the secondary antibody, applied carefully and left in the dark for 30 min. Subsequently, the sections were stained, first with diaminobenzidine and then with hematoxylin. The brown regions within the renal tissue were considered indicative of positive expression; each image was carefully examined and photographed. For quantification, positive area measurement was performed using ImageJ software (NIH, USA) in five random fields per section (400×).

### Immunofluorescence analysis

Frozen sections of kidney tissue were first fixed in 4% paraformaldehyde, followed by a 30-min room temperature incubation with 3% Triton. Non-specific binding was blocked by treating the sections with 5% BSA. Sections were incubated overnight at 4 °C with primary antibodies against CaSR and AQP1, washed in PBS, and exposed to goat anti-mouse Alexa Fluor 488 and goat anti-rabbit Alexa Fluor 555 secondary antibodies for 1 h at 37 °C. After PBS rinses, nuclei were counterstained with 4′,6-diamidino-2-phenylindole (DAPI) (5 min). Images were captured on a confocal laser-scanning microscope. For quantification, intensity quantification (mean fluorescence intensity, MFI) of CaSR was performed using ImageJ software in five random fields per section (400×). For colocalization analysis, images were analyzed using JACoP plugin in Fiji (ImageJ). Manual thresholds were set at 31 for the CaSR channel and 16 for the AQP1 channel for all images. Colocalization was quantified using Pearson’s correlation coefficient and Manders’ overlap coefficients (M1 and M2), where M1 represents the fraction of CaSR signal overlapping with AQP1, and M2 represents the fraction of AQP1 signal overlapping with CaSR.

### Quantitative real-time PCR

Kidney tissue samples were processed to isolate total RNA, which was then depleted of gDNA and converted into cDNA with a commercially available kit (AG11728). Next, qPCR was carried out to analyze the expression levels of inflammatory markers. The reactions utilized SYBR Green dye (AG11718) along with gene-specific primers targeting the inflammatory factors. The 2^−ΔΔCT^ method was employed to quantify mRNA expression levels, with β-Actin serving as the internal normalization control. The stability of β-actin as a housekeeping gene was validated by comparing Ct values across all treatment groups. One-way analysis of variance (ANOVA) revealed no significant differences in β-actin Ct values among groups (*p* > 0.05), confirming its suitability as an internal control (Supplementary Table S1). As shown in Table 1, all primer sequences were commercially synthesized by Sangon Biotech (Shanghai, China).

**TABLE 1:** Primer sequences for qRT-PCR.

Gene	Forward sequence (5’→3′)	Reverse sequence (5’→3′)
KIM-1	ACA​TAT​CGT​GGA​ATC​ACA​ACG​AC	ACA​AGC​AGA​AGA​TGG​GCA​TTG
TNF-α	ACG​GCA​TGG​ATC​TCA​AAG​AC	GTG​GGT​GAG​GAG​CAC​GTA​GT
MCP-1	CCA​CTC​ACC​TGC​TGC​TAC​TCA​TTC	CTG​CTG​CTG​GTG​ATC​CTC​TTG​TAG
IL-1β	TCG​CAG​CAG​CAC​ATC​AAC​AAG​AG	AGG​TCC​ACG​GGA​AAG​ACA​CAG​G
β-Actin	GGT​GGG​AAT​GGG​TCA​GAA​GG	GTA​CAT​GGC​TGG​GGT​GTT​GA

### Preparation of WLS-containing serum

Male Sprague-Dawley rats (180 ± 20 g, SPF grade) were obtained from the Guangdong Medical Laboratory Animal Center (License No. SCXK (Yue) 2019-0035). All animal procedures were approved by the Animal Ethics Committee of Guangzhou University of Chinese Medicine (Approval No. ZYD-2022-104).

Rats were orally administered WLS extract at a high dose of 6.48 g/kg (crude herb equivalent) once daily for 7 days. Based on the body surface area-based dose conversion method, the rat equivalent dose calculated from the human clinical dose (0.2571 g/kg/day) is 1.62 g/kg/day (conversion factor for rats: 6.3). A 4-fold scaling was then applied to obtain the high dose (6.48 g/kg) for serum preparation, ensuring sufficient serum concentrations of bioactive compounds for *in vitro* cell assays. Seven hours after the final administration, blood was collected via abdominal aortic puncture, allowed to clot for 1 h at room temperature, and centrifuged at 3500 rpm for 15 min. The serum was heat-inactivated at 56 °C for 30 min, filtered through a 0.22 μm filter, aliquoted, and stored at −80 °C. For cell experiments, mRTECs were treated with medium containing 2.5%, 5%, 10%, or 15% WLS-containing serum or control serum.

### Cell experiment

Mouse renal tubular epithelial cells (mRTECs, Cat# M4100-57) were purchased from a commercial source (ScienCell Research Laboratories, San Diego, CA, USA) and cultured in RPMI 1640 medium containing 10% fetal bovine serum (FBS) and 1% penicillin-streptomycin. Cultures were maintained at 37 °C under 5% CO_2_ atmosphere. To assess viability by Cell Counting Kit-8 (CCK-8) assay, mRTECs plated in 96-well plates were exposed to cisplatin at graded concentrations (2.5–80 μM) or WLS-containing serum (2%–15%). After pretreatment with WLS-containing serum (5% and 10%) for 24 h, mRTECs were treated with 10 µM cisplatin for 24 h.

### TUNEL staining

For frozen sections of kidney tissue, all fixed samples were incubated with TUNEL solution, strictly adhering to the protocol outlined in the One Step TUNEL Apoptosis Assay Kit’s instruction manual (Beyotime, CHN, C1086) for 1 h at 37 °C. Subsequently, DAPI staining of the sample was performed for 5 min, then observed and photographed by light microscopy within a day. For quantification, the percentage of TUNEL-positive cells was calculated by counting TUNEL-positive nuclei relative to total DAPI-stained nuclei in five random fields per section (400×) using ImageJ software.

### Annexin V/PI staining and flow cytometry

After treatment, mRTECs were trypsinized, washed twice with cold PBS, and resuspended in 1× Annexin V binding buffer at a density of 1 × 10^6^ cells/mL. Cells were stained with 5 μL Annexin V-fluorescein isothiocyanate (FITC) and 5 μL propidium iodide (PI) (YEASEN Biotech Co., Ltd, 40302ES60) for 15 min at room temperature in the dark, then analyzed within 1 h. Flow cytometry was performed using a BD Accuri C5 flow cytometer (BD Biosciences, USA) equipped with a 488 nm argon laser. Annexin V-FITC was detected in FL1 (530 nm) and PI in FL2 (585 nm). Compensation was adjusted using single-stained control samples. The gating strategy was as follows: Cells were first gated on FSC-A/SSC-A to exclude debris. Doublets were excluded by gating on FSC-A/FSC-H to select single cells. A total of 10,000 events were acquired from the single-cell gate. Early apoptotic cells were defined as Annexin V+/PI-, late apoptotic/necrotic cells as Annexin V+/PI+, and live cells as Annexin V-/PI-. Data were analyzed using FlowJo v10 (TreeStar, USA).

### ROS detection

For mRTECs, cells were cultured in 6-well plates at a density of 1.5 × 10^4^ cells per well. After treatment with cisplatin or WLS-containing serum, mRTECs were incubated with 2′,7′-dichlorodihydrofluorescein diacetate (DCFH-DA) solution (Beyotime, S0033S) and intracellular ROS was detected by flow cytometry.

### Western blot analysis

Total protein from mouse kidneys and mRTECs was quantified with a bicinchoninic acid (BCA) kit (Beyotime, P0011). Equal amounts were separated by SDS-PAGE and transferred to polyvinylidene difluoride (PVDF) membranes. Membranes were blocked, incubated with primary antibodies overnight (4 °C), followed by HRP-conjugated secondary antibodies for 1 h at room temperature. Protein bands were visualized with the Tanon 5200 system and quantified with ImageJ. All Western blot experiments were performed with three biological replicates.

### Fluo-4 AM measurements of intracellular Ca^2+^

mRTECs were seeded in 96-well plates (5 × 10^3^ cells per well). After 12 h of pre-treatment with WLS containing serum, mRTECs were exposed to cisplatin for an additional 12 h. Following PBS washes, cells were loaded with 100 µL Fluo-4 AM working solution (Beyotime, S1061S) and incubated at 37 °C for 30 min. Fluorescence was recorded at 490 nm excitation/525 nm emission using a plate reader. After fluorescence measurement, cells were lysed and total protein content was quantified using a BCA protein assay kit (Beyotime, China, P0011). Fluorescence values were normalized to protein content (fluorescence/mg protein) for each well.

### Molecular docking

The binding affinity of 15 WLS compounds to the CaSR (UniProt: Q2F3K6) was assessed by molecular docking. The CaSR structure was downloaded from the Protein Data Bank (PDB) database (https://www.rcsb.org), and ligand structures came from the PubChem database. Before docking, the protein was prepared by removing water and ligands, followed by energy minimization [OPLS2011 force field, convergence at RMS gradient <0.1 kcal/(mol·Å)]. Semi-flexible docking was performed using AutoDock Vina, and results were analyzed in PyMOL. To verify the docking protocol, two known CaSR ligands, NPS-2143 (antagonist) and cinacalcet (agonist), were docked into the CaSR binding pocket as positive controls. A binding energy threshold of ≤−5 kcal/mol was used to identify promising compounds.

### Statistical analysis

The statistical analysis was performed using SPSS 20.0, with results presented as mean values ±standard deviation. Normality (Shapiro-Wilk) and homogeneity of variances (Levene’s test) were tested before multiple comparisons. For multiple comparisons: when data were normally distributed with equal variances, one-way ANOVA followed by LSD *post hoc* test was used, with comparisons limited to pre-specified pairs (CP group vs. each treatment group); when data were normally distributed with unequal variances, Welch’s ANOVA followed by Dunnett’s T3 *post hoc* test was used; when data were not normally distributed, the Kruskal-Wallis test followed by Dunn-Bonferroni *post hoc* test was used (detailed in Supplementary Table S2). Statistical significance was defined as a *p*-value of less than 0.05. All outcome assessments, including histological scoring, TUNEL quantification, and immunofluorescence analysis, were performed by investigators blinded to group allocation. Sample sizes (*n* = 6 for animal studies, *n* = 3 for Western blot experiments) were determined based on our group’s preliminary experiments and long-term experience with the cisplatin-induced AKI model.

## Results

### Chemical composition analysis and identification of WLS

**FIGURE 1:**
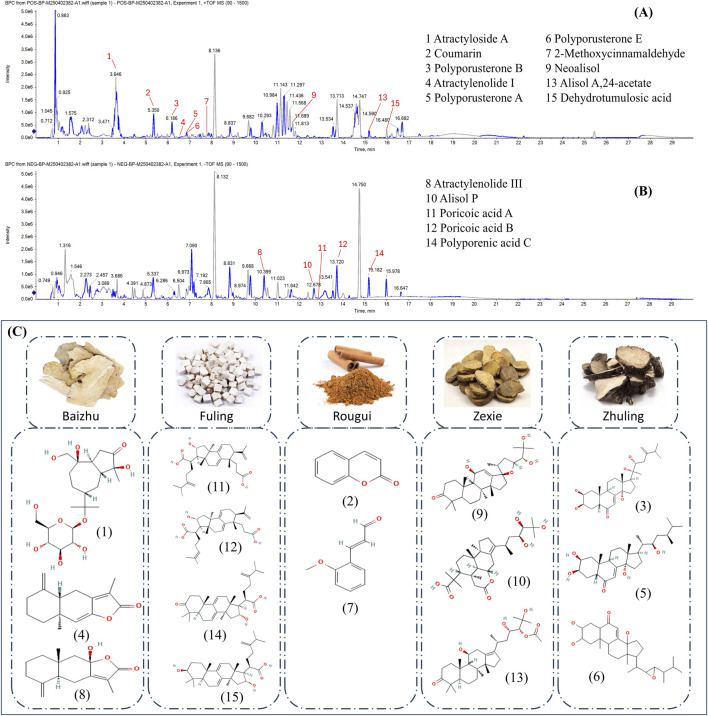
UHPLC-QE-MS analysis of components in WLS. **(A)** Positive ion mode. **(B)** Negative ion mode. **(C)** Structural formulas and sources of 15 compounds.

The chemical composition of the aqueous extract of WLS was qualitatively analyzed by UHPLC-QE-MS in positive and negative ion modes, respectively, and the total ion chromatograms were obtained (Figure 1). Based on the retention time, MS/MS fragmentation pattern, accurate molecular mass (mass deviation <5 ppm), and comparisons with previously reported data or public databases (e.g., MassBank, HMDB), the main components in WLS were identified. In total, 15 components were characterized and are shown in Table 2.

**TABLE 2:** Characterization of the chemical components of WLS by UHPLC-QE-MS.

No.	*t*_R_ (min)	Formula	Ion mode	Target mass (m/z)	Error (ppm)	MS^2^ fragments	Compounds
1	3.65	C_21_H_36_O_10_	[M+Na]^+^	471.21899	2.20704	120.07991, 176.10568, 221.12726, 223.10474, 231.10693, 453.21094, 471.22449	Atractyloside A
2	5.35	C_9_H_6_O_2_	[M+H]^+^	147.04399	0.61206	51.02310, 63.02242, 65.03921, 77.03811, 90.04418, 91.05452, 118.05962, 119.07526, 147.04410	Coumarin
3	6.19	C_28_H_44_O_6_	[M+H]^+^	477.31961	2.68163	135.11330, 147.07784, 253.15715, 301.18213, 405.27521, 413.29404	Polyporusterone B
4	6.55	C_15_H_18_O_2_	[M+H]^+^	231.13782	0.73549	231.13847, 163.07601, 105.07036, 77.03973	Atractylenolide I
5	6.85	C_28_H_46_O_6_	[M+H]^+^	479.33725	0.95966	109.06329, 135.11337, 147.08131, 213.12068, 231.12997, 255.17648, 283.16983, 301.17499, 479.33273	Polyporusterone A
6	6.88	C_28_H_44_O_5_	[M+H]^+^	461.32465	2.70958	191.09726, 225.12854, 265.15750, 283.16989, 301.17749, 303.18167, 311.19553, 329.21182, 425.29922	Polyporusterone E
7	7.70	C_10_H_10_O_2_	[M+H]^+^	163.07501	2.26889	51.02209, 65.03806, 77.03686, 91.05317, 103.05365, 115.05386, 121.06397, 131.04591, 135.07930	2-Methoxycinnamaldehyde
8	10.40	C_15_H_20_O_3_	[M-H]^-^	247.13326	2.71108	77.02020, 105.08297, 121.06525, 133.06502, 147.08266, 159.07832, 175.11398, 187.11310, 201.12590	Atractylenolide III
9	11.64	C_30_H_48_O_5_	[M+H]^+^	489.35498	4.92483	105.06672, 147.11549, 189.15958, 315.24774, 393.30090, 407.32275, 425.33429, 489.25980	Neoalisol
10	12.68	C_30_H_48_O_7_	[M-H]^-^	519.33203	2.23363	297.21991, 309.22006, 355.26215, 393.26978, 411.27432, 435.32437, 471.34445, 519.33325	Alisol P
11	12.89	C_31_H_46_O_5_	[M-H]^-^	497.32629	1.84989	411.29959, 437.34613, 455.32733, 479.31406	Poricoic acid A
12	13.72	C_30_H_44_O_5_	[M-H]^-^	483.30954	4.28295	435.29990, 453.30298, 483.30914	Poricoic acid B
13	15.18	C_32_H_52_O_6_	[M+Na]^+^	555.36536	0.43215	437.30600, 477.34528, 495.34052, 537.36737, 553.70099, 556.38086	Alisol A,24-acetate
14	15.20	C_31_H_46_O_4_	[M-H]^-^	481.33011	4.57065	245.03758, 481.35245	Polyporenic acid C
15	15.94	C_31_H_48_O_4_	[M+Na]^+^	507.34396	0.90668	227.19771, 507.29871	Dehydrotumulosic acid

### WLS alleviated renal dysfunction in cisplatin-induced AKI mice

**FIGURE 2:**
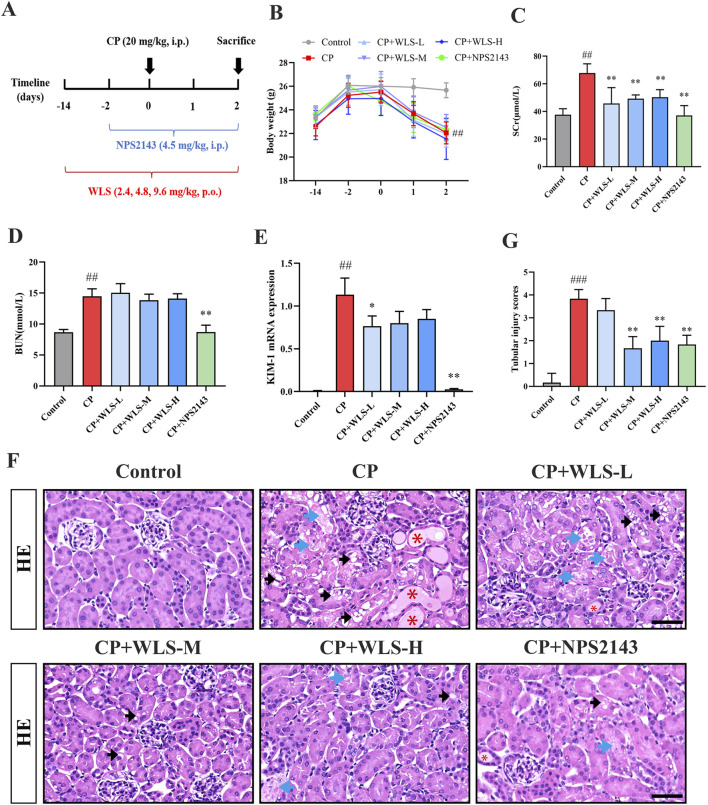
WLS alleviated renal dysfunction in cisplatin-induced AKI mice. The AKI model mice were induced by cisplatin (20 mg/kg) for 48 h, and administered with or without WLS (po.) at different doses and NPS2143 (4.5 mg/kg, i.p.). **(A)** The diagram of animal experiment (*n* = 6). **(B)** Body weight changes of mice during the experimental period (*n* = 6). **(C,D)** SCr and BUN levels in different groups of mice (*n* = 6). **(E)** mRNA expressions of KIM-1 in kidney tissues (*n* = 6). **(F,G)** Representative images of H&E staining and tubular injury scores (×400, scale bar = 50 μm, *n* = 6). Data were represented as mean ± standard deviation. ^##^ *p* < 0.01 vs. Control group. ^*^ *p* < 0.05, ^**^ *p* < 0.01 vs. CP group.

No mortality occurred in any group during the entire experimental period. As shown in Figure 2B, cisplatin treatment caused a significant body weight loss in all cisplatin-treated groups compared with the control group. No significant difference in body weight was observed between the CP group and WLS-treated groups. As shown in Figures 2C–E, the cisplatin-treated AKI mice exhibited markedly elevated SCr and BUN levels along with a substantial upregulation of KIM-1 mRNA expression when compared to healthy controls. These findings demonstrated that the mice in the model group had abnormal kidney function. Compared to the model group, the WLS and NPS2143 group exhibited significant attenuation in the expression levels of these biomarkers. Next, histological staining results showed that cisplatin-induced AKI mice had obvious tubular epithelial cell vacuolation, structural destruction, cell necrosis and protein casts, which were reduced by WLS or NPS2143 treatment (Figures 2F,G). These data indicated that cisplatin caused renal dysfunction, while WLS or NPS2143 treatment effectively improved renal function in cisplatin-induced AKI mice.

### WLS reduced renal inflammation, oxidative stress and apoptosis in cisplatin-induced AKI mice

**FIGURE 3:**
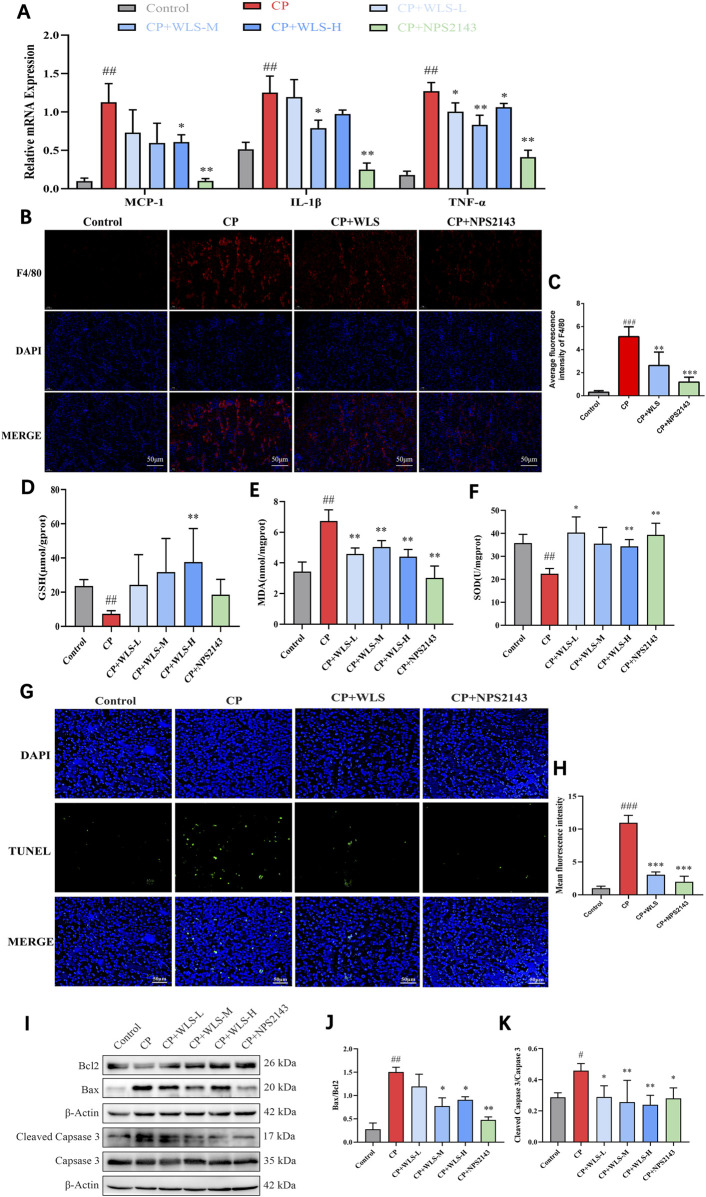
WLS reduced renal inflammation, oxidative stress, and apoptosis in cisplatin-induced AKI mice. **(A)** Relative mRNA expressions of MCP-1, IL-1β and TNF-α in kidney tissues (*n* = 6). **(B,C)** Representative immunofluorescence images and quantification of F4/80-positive cells (green) in kidney tissues (×400, scale bar = 50 μm, *n* = 6). **(D–F)** The levels of GSH, MDA and SOD in kidney tissues (*n* = 6). **(G,H)** Mean fluorescence intensity and representative images of TUNEL staining in kidney tissues (×400, scale bar = 50 μm, *n* = 6). **(I–K)** Protein expressions of Bcl2, Bax, Cleaved Caspase 3, and Caspase 3 in kidney tissues (*n* = 3). Data were represented as mean ± standard deviation. ^#^ *p* < 0.05, ^##^ *p* < 0.01 vs. Control group. ^*^ *p* < 0.05, ^**^ *p* < 0.01 vs. CP group.

When inflammation occurs in the kidney, it stimulates the renal resident cells or inflammatory cells to produce numerous pro-inflammatory factors, thereby exacerbating kidney damage. As shown in Figure 3A, qPCR analysis of renal inflammatory factors revealed markedly elevated MCP-1, TNF-α, and IL-1β mRNA levels in the model group versus the normal group, while different concentrations of WLS and NPS2143 reversed the upregulated pro-inflammatory cytokine expression. We next examined macrophage infiltration using F4/80 immunofluorescence staining. As shown in Figures 3B,C, cisplatin treatment significantly increased the number of F4/80-positive cells in kidney tissues compared with the Control group, indicating macrophage infiltration. WLS treatment significantly reduced the number of F4/80-positive cells compared with the CP group. In addition, the levels of oxidative stress-related markers (GSH and SOD) were reduced, while MDA was increased in the cisplatin-injured AKI mice (Figures 3D–F). Notably, WLS and NPS2143 treatment attenuated oxidative stress in AKI mice. To verify whether apoptosis was involved in kidney injury, TUNEL staining was performed in the mouse kidney sections. AKI mice treated with WLS and NPS2143 exhibited reduced TUNEL fluorescence (Figures 3G,H). Furthermore, as shown in Figures 3I–K, analysis of apoptosis-related proteins revealed that both WLS and NPS2143 attenuated the downregulation of Bcl2 and the elevation of Bax and cleaved Caspase 3 triggered by cisplatin. This suggests that WLS and NPS2143 reduced inflammation, attenuated oxidative stress, and prevented apoptosis in mice with cisplatin-induced AKI.

### WLS-containing serum inhibited cisplatin-induced ROS and apoptosis in mRTECs

**FIGURE 4:**
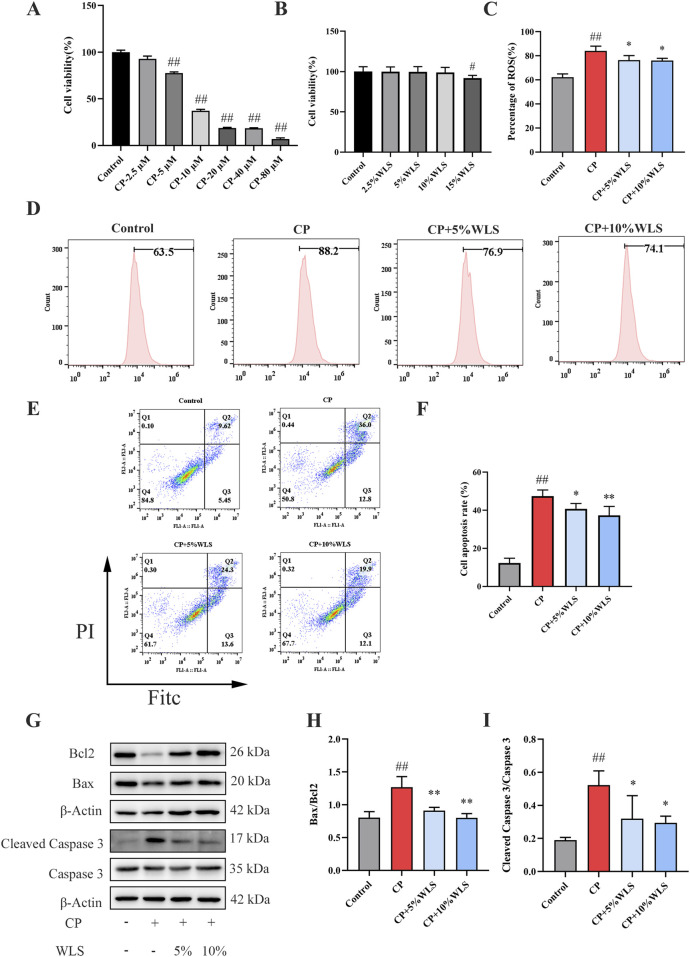
WLS-containing serum inhibited cisplatin-induced ROS and apoptosis in mRTECs. **(A,B)** mRTECs were exposed to different concentrations of cisplatin (2.5, 5, 10, 20, 40, and 80 µM) or WLS-containing serum (2.5%, 5%, 10%, and 15%) to detect the cell viability by CCK8 kit (*n* = 6). **(C,D)** The ROS levels of cisplatin-induced mRTECs were detected by flow cytometry (*n* = 6). **(E,F)** The apoptosis rate of cisplatin-induced mRTECs was detected by flow cytometry (*n* = 6). **(G–I)** Protein expressions of Bcl2, Bax, Cleaved Caspase 3, and Caspase 3 in mRTECs. Data were represented as mean ± standard deviation (*n* = 3). ^#^ *p* < 0.05, ^##^ *p* < 0.01 vs. Control group. ^*^ *p* < 0.05, ^**^ *p* < 0.01 vs. CP group.

To clarify the modeling concentration of cisplatin, mRTECs were treated with concentrations ranging from 2.5 to 80 μM for 24 h and cell viability was measured. As shown in Figure 4A, the viability of the cells was approximately 50% when the concentration of cisplatin was 10 μM, indicating that this was appropriate modeling concentration. mRTECs were also stimulated with different concentrations of WLS-containing serum for 24 h to test the effect on cell viability. The results showed that 2.5%, 5% and 10% serum did not affect the cells, while 15% had some toxicity to the cells; therefore, 5% and 10% were chosen as the administered concentrations of WLS (Figure 4B). Next, the effect of WLS-containing serum on ROS and apoptosis in cisplatin-induced injury of mRTECs was assessed using flow cytometry. As shown in Figures 4C–F, ROS and apoptosis levels in mRTECs were elevated after cisplatin-induced injury, but intervention with WLS-containing serum intervention reduced their levels. Furthermore, Western blotting to assess apoptotic protein expression, WLS treatment attenuated cisplatin-induced damage in mRTECs by lowering the Bax/Bcl-2 and Cleaved Caspase 3 levels (Figures 4G–I). The findings indicated that cisplatin increased ROS levels and apoptosis in mRTECs, effects that were attenuated by WLS.

### WLS inhibited the CaSR/CaMKKβ/AMPK signaling in cisplatin-induced AKI mice

**FIGURE 5:**
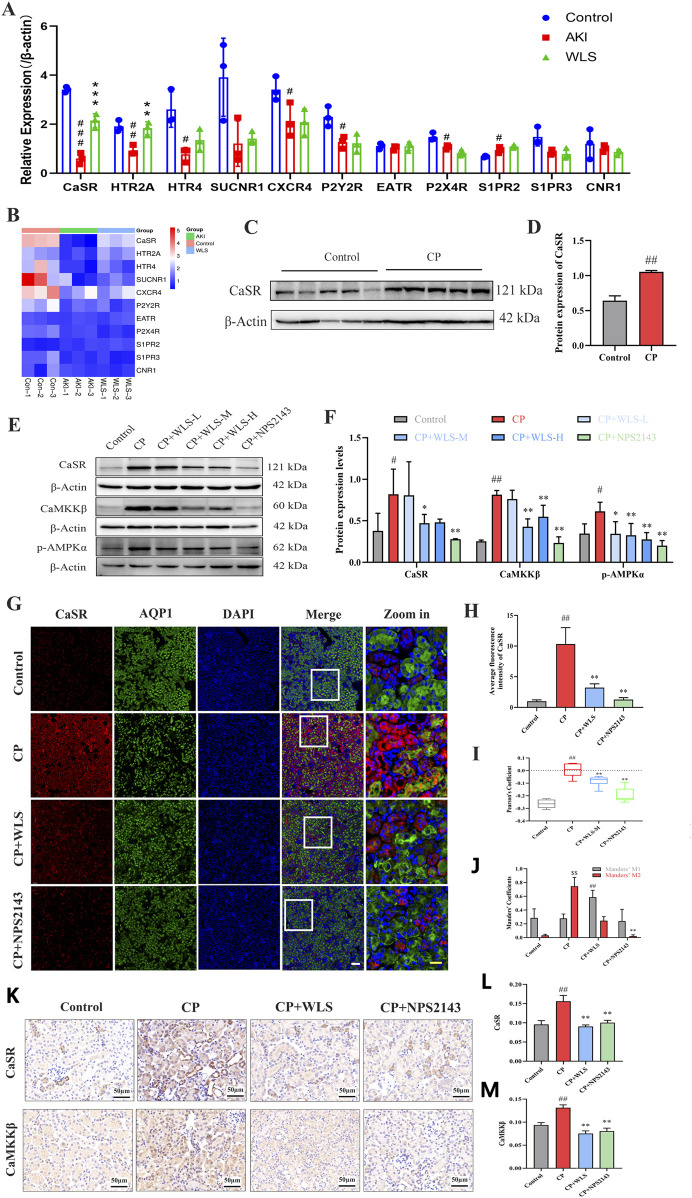
WLS inhibited CaSR/CaMKKβ/AMPK pathway in cisplatin-induced AKI mice. **(A)** mRNA expression levels of 11 kidney-expressed GPCRs screened by RT-qPCR (*n* = 3). **(B)** Heatmap of the screening results. **(C,D)** Protein expressions of CaSR in kidney tissues (*n* = 5). **(E,F)** Protein expression of CaSR, CaMKKβ, and p-AMPK in kidney tissues (*n* = 3). **(G–J)** Immunofluorescence analysis of CaSR and AQP1 in kidney tissues (×400, scale bar = 50 μm, *n* = 6). **(G)** Representative images of CaSR (red), AQP1 (green), and DAPI (blue). **(H)** Mean fluorescence intensity of CaSR. **(I)** Pearson’s correlation coefficients for CaSR and AQP1 colocalization. **(J)** Manders’ overlap coefficients (M1: CaSR overlapping with AQP1; M2: AQP1 overlapping with CaSR). **(K–M)** Representative images and quantification of CaSR and CaMKKβ in kidney tissues were detected by immumohistochemical staining (×400, scale bar = 50 μm, *n* = 6). Data were represented as mean ± standard deviation. ^#^ *p* < 0.05, ^##^ *p* < 0.01 vs. Control group, ^*^ *p* < 0.05, ^**^ *p* < 0.01 vs. CP group.

To identify which GPCRs are regulated by WLS in cisplatin-induced AKI, we examined the mRNA expression of 11 GPCRs reported to be expressed in kidney tissues. As shown in Figure 5A, cisplatin treatment significantly downregulated 7 GPCRs (CaSR, HTR2A, HTR4, CXCR4, P2Y2R, P2X4R, and S1PR2) compared with the Control group. Compared with the CP group, WLS treatment significantly upregulated only two GPCRs: CaSR and HTR2A. Among these, CaSR showed the most significant regulation (Figure 5B). Therefore, CaSR was selected for subsequent mechanistic studies. We next examined CaSR protein expression in kidney tissues. As shown in Figures 5C,D, CaSR protein expression was significantly increased in the CP group compared with the Control group. We then examined the protein levels of CaSR and its downstream effectors CaMKKβ and p-AMPK. As shown in Figures 5E,F, the expression of CaSR, CaMKKβ, and p-AMPK was upregulated in the CP group, while treatment with WLS or NPS2143 downregulated their expression, except for the low-dose WLS group. In addition, immunofluorescence analysis showed markedly elevated CaSR expression in the model group, which co-localized with the proximal renal tubular marker AQP1 (Figure 5G). Quantitative analysis revealed that WLS treatment significantly reduced CaSR mean fluorescence intensity (Figure 5H). Colocalization analysis using Pearson’s correlation coefficient and Manders’ overlap coefficients showed that Pearson’s r values were low across all groups (Figure 5I). However, Manders’ M2 was markedly increased in the CP group (0.745 ± 0.132) compared with Control (0.032 ± 0.016), indicating abnormal colocalization of AQP1 with CaSR after injury. WLS treatment significantly reduced M2 to 0.244 ± 0.058 and increased M1 to 0.587 ± 0.100, suggesting restoration of normal colocalization patterns (Figure 5J). Immunohistochemical results also showed that positive expression of CaSR and CaMKKβ was increased in renal tissues of mice in the AKI model group, and this expression was downregulated after administration of WLS or NPS2143 treatment (Figures 5K–M). These results suggested that cisplatin induced activation of the CaSR/CaMKKβ/AMPK pathway, while WLS or NPS2143 served to inhibit this pathway.

### WLS-containing serum inhibited cisplatin-induced apoptosis in mRTECs was attenuated after activation with CaSR

**FIGURE 6:**
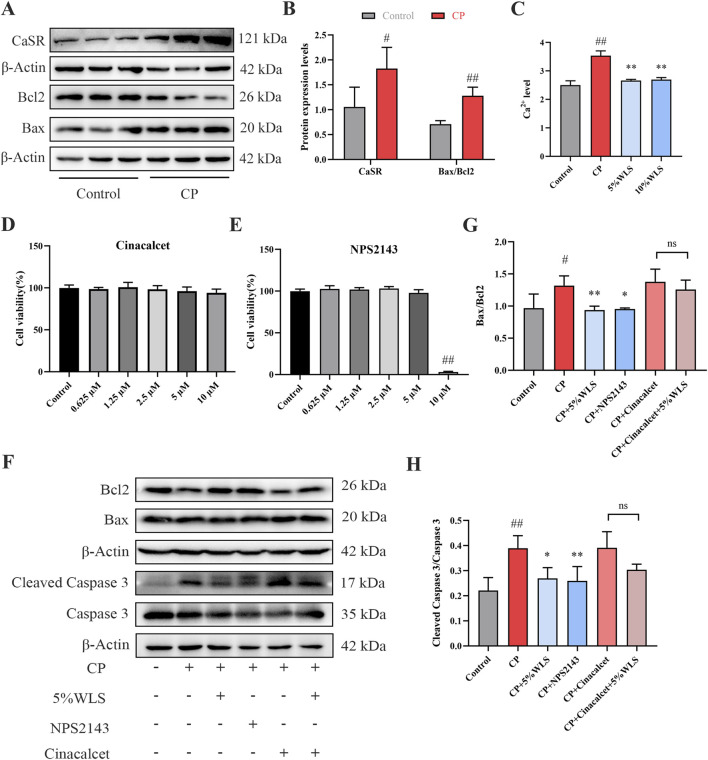
The inhibition of cisplatin-induced apoptosis by WLS-containing serum was attenuated after activation of the CaSR in mRTECs. The mRTECs were pre-treated with or without 5% WLS-containing serum, NPS2143 (CaSR inhibitor, 2.5 µM), or cinacalcet (CaSR agonist, 2.5 µM) for 12 h followed stimulated by cisplatin (10 µM) for 12 h **(A,B)** Protein expressions of CaSR, Bcl2, and Bax (*n* = 3). **(C)** The Ca^2+^ levels in mRTECs were determined by Fluo-4 calcium assay kit and normalized to total protein content (*n* = 3). **(D,E)** mRTECs were exposed to NPS2143 (0.625, 1.25, 2.5, 5, and 10 µM) or cinacalcet (0.625, 1.25, 2.5, 5, and 10 µM) to detect the cell viability by CCK8 kit (*n* = 6). **(F–H)** Protein expressions of Bcl2, Bax, Cleaved Caspase 3, and Caspase 3 (*n* = 3). Data were represented as mean ± standard deviation. ^#^ *p* < 0.05, ^##^ *p* < 0.01 vs. Control group, ^*^ *p* < 0.05, ^**^ *p* < 0.01 vs. CP group, ns, not significant.

As shown in Figures 6A,B, CaSR and Bax/Bcl2 protein expression levels were increased after 12 h incubation with 10 μM cisplatin, indicating that CaSR may be involved in the process of apoptosis in mRTECs cells induced by cisplatin. CaSR is known to regulate intracellular Ca^2+^ levels by sensing changes in calcium concentration. Therefore, intracellular Ca^2+^ levels in cisplatin-treated cells were measured after WLS-containing serum treatment to assess whether WLS is involved in the regulation of Ca^2+^ concentration. Intracellular Ca^2+^ was significantly elevated in the cisplatin-induced injury cell model, but decreased when 5% and 10% WLS-containing serum was added (Figure 6C). To further investigate the role of CaSR in apoptosis, the CaSR inhibitor (NPS2143) and agonist (cinacalcet) were used for intervention experiments. The mRTECs viability results showed that concentrations of NPS2143 and cinacalcet at 0–5 µM were non-toxic to cells (Figures 6D,E). Therefore, a final concentration of 2.5 µM for each was selected for treatment. As shown in Figures 6F–H, cisplatin-induced elevations in the Bax/Bcl-2 ratio and cleaved caspase-3 protein expression in mRTECs were reduced by treatment with 5% WLS or NPS2143, but treatment with cinacalcet alone did not reduce these levels. Furthermore, the reduction in apoptosis-related protein levels in the 5% WLS plus cinacalcet group was not statistically significant compared to the cinacalcet alone group. These results suggested that WLS-containing serum may inhibit cisplatin-induced apoptosis in mRTECs by modulating CaSR.

### WLS-containing serum inhibited the CaSR/CaMKKβ/AMPK signaling in cisplatin-induced mRTECs injury

**FIGURE 7:**
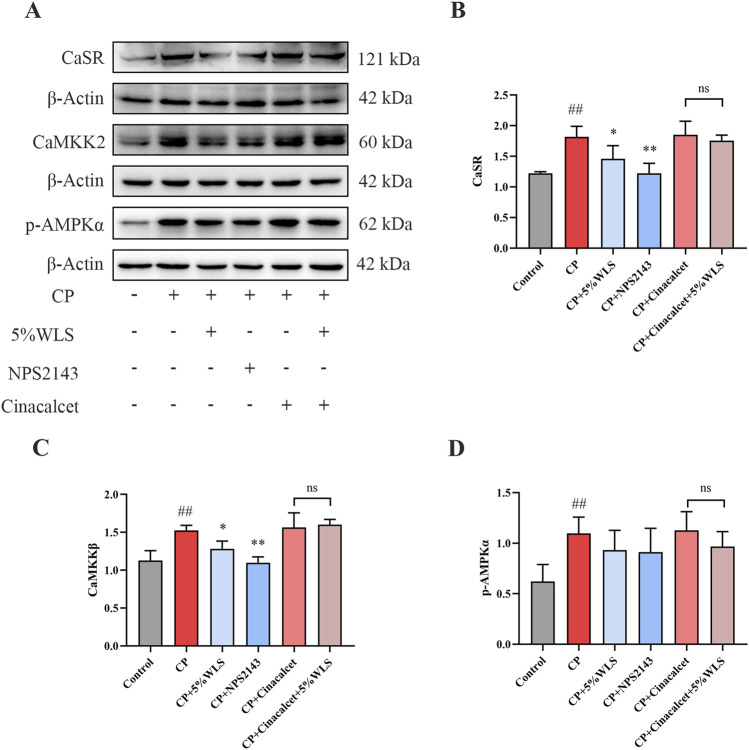
WLS-containing serum ameliorated cisplatin-induced apoptosis via inhibiting CaSR/CaMKKβ/AMPK pathway in mRTECs. **(A–D)** Protein expressions of CaSR, CaMKKβ, and p-AMPK. Data were represented as mean ± standard deviation (*n* = 3). ^##^ *p* < 0.01 vs. Control group, ^*^ *p* < 0.05, ^**^ *p* < 0.01 vs. CP group, ns, not significant.

To further validate the role of WLS-containing serum in modulating the CaSR/CaMKKβ/AMPK pathway, alterations in protein expression of pathway components were assessed after treatment with CaSR inhibitors or agonists alone or in combination with WLS. As shown in Figure 7, the cisplatin-induced increases in CaSR, CaMKKβ and p-AMPK protein expression levels in mRTECs were reduced by treatment with 5% WLS or NPS2143, but treatment with cinacalcet did not reduce these levels. Furthermore, compared to the cinacalcet alone group, the 5% WLS plus cinacalcet group did not show reduced protein expression levels of the CaSR/CaMKKβ/AMPK pathway, indicating that cinacalcet reversed the efficacy of WLS. This evidence suggested that WLS may exert its effects by regulating the CaSR/CaMKKβ/AMPK pathway.

### Molecular docking between representative components and CaSR

**FIGURE 8:**
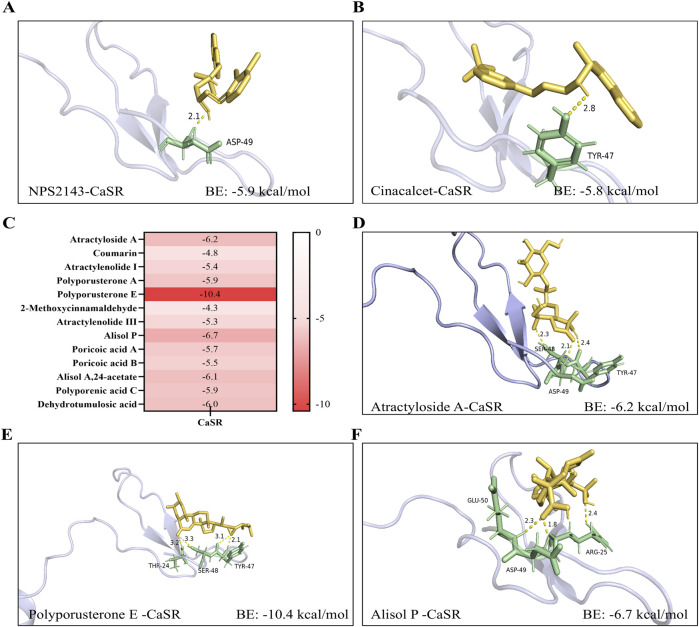
Molecular docking between representative components and CaSR. **(A,B)** Docking scores of positive controls (NPS2143 and Cinacalcet). **(C)** Docking scores of 15 WLS components. **(D–F)** Top 3 WLS components with best binding affinity to CaSR (Atractyloside A, Polyporusterone E, and Alisol P, respectively).

To further investigate the affinity of the identified components in WLS for the CaSR binding site, molecular docking simulations were performed on the 15 characterized constituents. As shown in Figures 8A,B, the positive controls NPS-2143 and cinacalcet exhibited binding affinities of −5.9 and −5.8 kcal/mol, respectively, confirming the reliability of the docking protocol. Among the 15 WLS components, 13 exhibited binding affinity for CaSR, and 11 of these had binding energies ≤ −5 kcal/mol, meeting the threshold for promising compounds (Figures 8C–F), with the top three being Polyporusterone E (−10.4 kcal/mol), Alisol P (−6.7 kcal/mol), and Atractyloside A (−6.2 kcal/mol). These results suggested that CaSR may be a potential target of WLS in the treatment of AKI.

## Discussion

AKI is a worldwide health concern linked to significant morbidity and mortality, affecting 10–15% of hospitalized patients and over 50% of ICU admissions (Cerda et al., 2026). Currently, managing AKI relies predominantly on symptomatic and supportive care, offering few therapeutic choices that are difficult to implement, especially for improving patients’ long-term outcomes. However, some newly discovered potential therapeutic targets offer new avenues for treatment (Li et al., 2023). Traditional Chinese medicine has shown certain advantages in the treatment of AKI, as evidenced by its therapeutic effects via a multi-target and multi-pathway approach, which helps to comprehensively regulate physiological functions, thereby alleviating the pathological process of AKI (Liu et al., 2023). Moreover, active components of traditional Chinese medicine, such as quercetin, luteolin, and icariin, have the potential to alleviate apoptosis in cisplatin-induced nephrotoxicity (Fang et al., 2021).

Derived from the “Treatise on Febrile Diseases” (Shang Han Lun), WLS represents a classical traditional formula with extensive clinical applications across multiple pathological conditions (Ahn et al., 2024). Identification of the components of the aqueous extract of WLS has been rarely reported in the literature. Therefore, we investigated the chemical basis of WLS by UHPLC-QE-MS. The analytical results indicated that the peaks were well separated and a total of 15 components were identified. The 15 identified components were subjected to molecular docking with CaSR. Thirteen showed binding affinity, with 11 having binding energies ≤ −5 kcal/mol. Polyporusterone E exhibited the strongest binding (−10.4 kcal/mol), notably higher than the positive controls NPS2143 (−5.9 kcal/mol) and cinacalcet (−5.8 kcal/mol), followed by Alisol P (−6.7 kcal/mol) and Atractyloside A (−6.2 kcal/mol). Consistent with these docking results, several components have reported renal activities. Atractylenolide III protects against cisplatin-induced AKI via the PIK3CA/AKT pathway, directly supporting our findings. Atractylenolide I and Poricoic acid A attenuate renal fibrosis (Guo et al., 2021; You et al., 2025; Zhao et al., 2024). For Polyporusterone E and Alisol P, direct renal evidence is limited, though their structural analogs (Polyporusterone B, Alisol B 23-acetate) exhibit anti-inflammatory or nephropathy-related activities (Chen et al., 2020; Song et al., 2025). In contrast, Atractyloside A has been reported to protect the intestinal mucosal barrier (Tu et al., 2020), suggesting a potential role in gut-kidney crosstalk. Thus, multiple components of WLS may contribute to CaSR modulation, with Polyporusterone E being the most promising candidate for future validation.

Cisplatin-induced nephrotoxicity is a widely used and pathologically well-characterized animal model for studying AKI; it can cause tubular injury and renal dysfunction (Lin et al., 2024). Our model group showed abnormalities in indices of renal function and renal pathology, along with increased markers of renal injury, indicating that the model was successfully established. In this study, we observed that WLS exhibited nephroprotective effects in cisplatin-induced AKI mice, leading to improvements in biochemical markers, markers of kidney injury, and pathological changes. Cisplatin-induced DNA damage triggers mitochondrial dysfunction and ROS generation, ultimately leading to tubular cell apoptosis and inflammation (Barnes et al., 2025; Tong et al., 2023). In the present study, WLS reduced MCP-1, TNF-α, and IL-1β expression, restored GSH/SOD, and decreased MDA levels, demonstrating its anti-inflammatory and antioxidant effects. Increased ROS can upregulate inflammatory factors and trigger apoptosis (Tian et al., 2026). Our previous studies have shown that WLS protects against kidney diseases by delaying cellular senescence (Liu et al., 2025) and ameliorating renal fibrosis (Zeng et al., 2026). It has also been reported that WLS restores gut microbiota homeostasis (Cai et al., 2023). The gut-kidney axis is recognized as a key pathway in kidney disease pathogenesis (Jin et al., 2026). In cisplatin-induced AKI, several traditional Chinese medicine (TCM) formulas exert renoprotective effects via this axis by modulating gut microbiota (Zhang et al., 2026; Zou et al., 2022). Furthermore, the “gut microbiota-inflammation-oxidative stress axis” has been identified as a key pathway linking gut dysbiosis to renal inflammation and oxidative stress (Chang et al., 2025; Li X. J. et al., 2024). Based on this evidence, we speculate that the anti-inflammatory and antioxidant effects of WLS may involve gut microbiota regulation via the gut-kidney axis, although this requires further validation. Consistently, the increased level of apoptosis was inhibited by WLS treatment in AKI mice. In addition, WLS-containing serum also alleviated apoptosis in mRTECs induced by cisplatin. These findings suggest that WLS has potential as a nephroprotective agent for cisplatin-induced AKI.

Our study advances beyond general observations of reduced apoptosis by identifying a specific pathway through which WLS acts. Screening of 11 kidney-expressed GPCRs identified CaSR as the most significantly regulated by WLS. Using the CaSR inhibitor NPS2143 and agonist cinacalcet, we demonstrated that the anti-apoptotic effect of WLS is functionally associated with CaSR, acting through the CaMKKβ/AMPK axis. These findings provide a mechanistic framework for understanding WLS nephroprotection. Numerous studies have shown that CaSR has significant physiological functions in the kidney, regulating apoptosis. CaSR may be activated or inhibited in different renal diseases. The activation of CaSR ameliorates nephrotoxicity and kidney damage stemming from elevated Ca^2+^ levels (Gu et al., 2020). On the contrary, CaSR activation has been associated with podocyte injury and proximal tubular cell apoptosis in certain pathological conditions. Renal calcium homeostasis protects against apoptosis, whereas dysregulated calcium signaling via transient receptor potential cation channel subfamily C member 6 (TRPC6) induces podocyte calcium overload, oxidative stress, and apoptosis (Patel et al., 2026). Interestingly, we found that CaSR was upregulated in cisplatin-induced AKI mice and intracellular Ca^2+^ levels were increased after treatment with cisplatin in mRTECs. CaMKKβ is a well-established downstream component in Ca^2+^ signaling. It can phosphorylate and stimulate the AMPK, and the CaMKKβ/AMPK signaling pathway has been demonstrated to regulate apoptosis in the kidney (Han et al., 2024). In this study, treatment with WLS or NPS2143 reduced the protein expression of CaSR and downstream CaMKKβ/AMPK signaling components. Next, cinacalcet, which is an agonist of CaSR, did not alleviate apoptosis in mRTECs after they were induced by cisplatin. Interestingly, treatment with cinacalcet combined with WLS-containing serum had no therapeutic effect and did not downregulate the CaSR/CaMKKβ/AMPK pathway. Based on these observations, we speculate that WLS reduces apoptosis via the CaSR/CaMKKβ/AMPK pathway. However, the upstream mechanisms regulating this pathway remain unclear. A recent review has highlighted that gut microbiota metabolic reprogramming can mediate distant organ injury through the gut-kidney axis by modulating lipid and amino acid metabolism and inflammatory pathways (Wang et al., 2026). Given that WLS restores gut microbiota homeostasis (Cai et al., 2023), we further speculate that WLS may regulate gut microbiota metabolic reprogramming, thereby influencing CaSR/CaMKKβ/AMPK activation and contributing to its anti-apoptotic effects. This speculation offers a new theoretical perspective for understanding the upstream mechanisms of WLS’s nephroprotective effects, although further experimental validation is required.

However, the interpretation of AMPK changes in our study requires further clarification. In our cisplatin-induced AKI model, cisplatin increased p-AMPK levels, while WLS treatment reduced p-AMPK and protected against AKI. Consistent with our findings, (Xing et al., 2019), reported that ginsenoside Rb3 protects against cisplatin-induced AKI by suppressing AMPK activation. In contrast, other studies have reported that AMPK activation contributes to renal protection in cisplatin-induced AKI (Park et al., 2022; Salah et al., 2025). Thus, both increased and decreased p-AMPK have been associated with renal protection, suggesting that AMPK changes may not be the primary protective mechanism but rather a downstream event. In our study, WLS protects primarily by inhibiting the CaSR/CaMKKβ pathway, and the reduction in p-AMPK likely reflects a downstream consequence of reduced upstream stress (Ca^2+^ overload and ROS). This interpretation is supported by (Li et al., 2020), who showed that the AMPK inhibitor Compound C protects against cisplatin-induced AKI through AMPK-independent mechanisms, indicating that AMPK changes can be an epiphenomenon. Thus, whether AMPK activation in our model is protective, detrimental, or simply an epiphenomenon remains to be determined. The precise role of AMPK requires further validation using AMPK-specific inhibitors or activators.

Several limitations of this study should be acknowledged. First, the UHPLC-QE-MS analysis was qualitative only; we did not quantify the absolute concentrations of the 15 identified compounds. Second, the protective effect of WLS was evaluated using a preventive protocol rather than a therapeutic protocol, which limits clinical translatability. Additionally, only a single cisplatin dose (20 mg/kg) and time point (48 h) were used; dose-response and time-course studies are needed to fully characterize the dynamic effects of WLS. Third, no established positive control drug is currently approved for cisplatin-induced AKI; therefore, such a comparator was not included. Future studies should evaluate WLS against standard supportive care. Fourth, we did not perform genetic manipulation (e.g., CaSR knockdown or knockout) to establish definitive causality, nor did we conduct intervention experiments using AMPK inhibitors/agonists to verify the functional role of AMPK in WLS-mediated protection. Fifth, WLS is a multi-component herbal formula, and the present study did not identify its specific active compounds. However, the docking results prioritized Polyporusterone E, Alisol P, and Atractyloside A as top CaSR-binding candidates, with Polyporusterone E showing the strongest affinity (−10.4 kcal/mol). Future validation of these compounds in the cisplatin-induced AKI model is warranted.

Despite these limitations, the present study identified the components in the aqueous extract of WLS by UHPLC-QE-MS and showed that WLS inhibits apoptosis in cisplatin-induced AKI in a manner associated with modulation of the CaSR/CaMKKβ/AMPK pathway. Future studies should evaluate WLS in a therapeutic protocol (administration after cisplatin) to better mimic clinical practice, particularly for the transition from acute to chronic kidney injury. In addition, future studies should identify which WLS components regulate CaSR and generate renal tubule-specific CaSR knockout mice to further clarify this target.

## Conclusion

**FIGURE 9:**
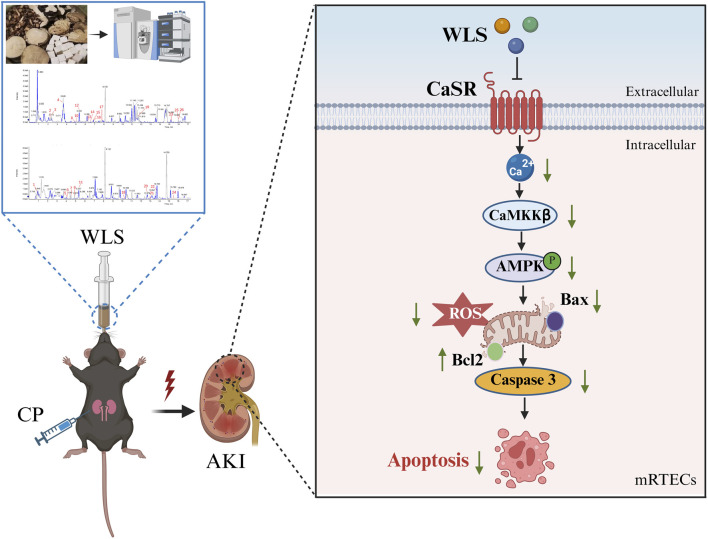
The graphic illustration of WLS protecting against AKI.

Overall, our study demonstrated that WLS effectively mitigated renal impairment and renal cell apoptosis in mice with cisplatin-induced AKI. The protective effect of WLS was associated with inhibition of apoptosis and modulation of the CaSR/CaMKKβ/AMPK pathway (Figure 9). In addition, 15 major active compounds in the aqueous extract of WLS were identified by UHPLC-QE-MS, providing the basis for elucidating its pharmacological effects. Thus, WLS may represent a promising candidate for therapeutic intervention in AKI.

## Data Availability

The original contributions presented in the study are included in the article/Supplementary Material, further inquiries can be directed to the corresponding author/s.
