# Supplementary material for: Wulingsan alleviates cisplatin-induced acute kidney injury and inhibits renal tubular epithelial cell apoptosis in association with the CaSR/CaMKKβ/AMPK pathway
Source: Front Pharmacol. 2026 Jun 26;17:1824226. doi: 10.3389/fphar.2026.1824226 (PMC13351979; doi:10.3389/fphar.2026.1824226)
Supplement: Supplementary file 1 [file Table1.docx]

**Supplementary Table S1 Validation of β-actin stability as a housekeeping gene across treatment groups (*n* = 6).**

| **Gene** | **Control** | **CP** | **CP+WLS-L** | **CP+WLS-M** | **CP+WLS-H** | **CP+NPS2143** |
| --- | --- | --- | --- | --- | --- | --- |
| **IL-1β** | 17.63 | 16.20 | 15.60 | 16.69 | 15.94 | 16.26 |
|  | 16.40 | 16.17 | 16.12 | 15.96 | 15.62 | 16.21 |
|  | 16.56 | 15.99 | 15.83 | 16.31 | 16.79 | 16.06 |
|  | 16.11 | 16.38 | 16.98 | 15.80 | 15.67 | 16.37 |
|  | 16.38 | 16.36 | 15.15 | 15.81 | 17.02 | 16.59 |
|  | 15.98 | 16.30 | 16.59 | 15.95 | 16.90 | 16.40 |
| Mean ± SD | 16.51±0.59 | 16.23±0.15 | 16.04±0.67 | 16.09±0.35 | 16.32±0.65 | 16.3±0.2 |
| **KIM-1** | 15.78 | 16.21 | 15.87 | 16.21 | 17.56 | 17.11 |
|  | 15.67 | 16.15 | 18.44 | 16.37 | 16.47 | 16.78 |
|  | 16.97 | 16.09 | 16.20 | 15.79 | 17.03 | 16.50 |
|  | 17.33 | 16.40 | 16.28 | 16.23 | 15.95 | 16.98 |
|  | 17.13 | 15.84 | 17.00 | 16.56 | 16.36 | 16.66 |
|  | 17.56 | 16.18 | 16.30 | 17.94 | 16.53 | 17.34 |
| Mean ± SD | 16.74±0.81 | 16.15±0.18 | 16.68±0.94 | 16.52±0.74 | 16.65±0.56 | 16.9±0.31 |
| **TNF-α** | 16.47 | 16.19 | 16.34 | 16.21 | 16.34 | 16.07 |
|  | 16.41 | 16.13 | 16.30 | 16.40 | 16.67 | 16.14 |
|  | 16.21 | 16.06 | 16.12 | 16.44 | 16.34 | 16.13 |
|  | 16.37 | 16.43 | 16.21 | 16.52 | 17.14 | 16.33 |
|  | 16.36 | 15.89 | 16.54 | 16.74 | 15.99 | 16.52 |
|  | 16.10 | 16.80 | 16.22 | 16.29 | 16.42 | 16.33 |
| Mean ± SD | 16.32±0.14 | 16.25±0.32 | 16.29±0.15 | 16.46±0.19 | 16.48±0.39 | 16.26±0.17 |
| **MCP-1** | 16.46 | 15.81 | 15.92 | 16.13 | 15.87 | 15.77 |
|  | 16.07 | 16.12 | 16.24 | 16.04 | 16.34 | 15.80 |
|  | 16.02 | 15.61 | 15.80 | 16.05 | 16.28 | 15.68 |
|  | 15.96 | 16.26 | 16.08 | 16.28 | 16.98 | 16.10 |
|  | 16.30 | 15.41 | 16.20 | 16.73 | 15.74 | 16.18 |
|  | 15.90 | 16.46 | 16.04 | 16.00 | 15.94 | 15.88 |
| Mean ± SD | 16.12±0.22 | 15.94±0.4 | 16.05±0.17 | 16.25±0.29 | 16.19±0.45 | 15.9±0.2 |

Note: One-way ANOVA revealed no significant differences in β-actin Ct values among groups for all four target genes (p > 0.05), confirming that β-actin expression was stable across all treatment groups.
