# Supplementary material for: Wulingsan alleviates cisplatin-induced acute kidney injury and inhibits renal tubular epithelial cell apoptosis in association with the CaSR/CaMKKβ/AMPK pathway
Source: Front Pharmacol. 2026 Jun 26;17:1824226. doi: 10.3389/fphar.2026.1824226 (PMC13351979; doi:10.3389/fphar.2026.1824226)
Supplement: Supplementary file 2 [file Table2.docx]

**Supplementary Table S2 Detailed statistical results for all figures.**

**Statistical results for Figure 2**

Table 2-1. Body weight changes in cisplatin-induced AKI mice（*n*=6）**（Fig. 1B）**

| Body weight | Group | Mean ± SD  （$\bar{x}\pm s$） | *Shapiro-Wilk* | | *levene test* | | *ANOVA* | | *LSD* |
| --- | --- | --- | --- | --- | --- | --- | --- | --- | --- |
|  |  |  | *W* | *P* | *F* | *P* | *F* | *P* | *P* |
|  | Control | 25.65±0.65 | 0.877 | 0.254 | 1.340 | 0.275 | 23.348 | 0.000 | - |
|  | CP | 22.05±0.93^##^ | 0.959 | 0.808 |  |  |  |  | 0.000 |
|  | CP+WLS-L | 22.22±0.92 | 0.953 | 0.767 |  |  |  |  | 0.780 |
|  | CP+WLS-M | 21.98±0.47 | 0.978 | 0.942 |  |  |  |  | 0.889 |
|  | CP+WLS-H | 21.25±0.61 | 0.832 | 0.112 |  |  |  |  | 0.101 |
|  | CP+NPS2143 | 21.58±0.69 | 0.846 | 0.147 |  |  |  |  | 0.332 |

Table 2-2. Serum creatinine levels in cisplatin-induced AKI mice（*n*=6）**（Fig. 2C）**

| SCr | Group | Mean ± SD  （$\bar{x}\pm s$） | *Shapiro-Wilk* | | *levene test* | | *ANOVA* | | *LSD* |
| --- | --- | --- | --- | --- | --- | --- | --- | --- | --- |
|  |  |  | *W* | *P* | *F* | *P* | *F* | *P* | *P* |
|  | Control | 37.72±4.26 | 0.963 | 0.840 | 1.390 | 0.256 | 16.080 | 0.000 | - |
|  | CP | 67.83±6.65^##^ | 0.978 | 0.942 |  |  |  |  | 0.000 |
|  | CP+WLS-L | 45.78±11.46^**^ | 0.915 | 0.471 |  |  |  |  | 0.000 |
|  | CP+WLS-M | 49.23±2.74^**^ | 0.803 | 0.063 |  |  |  |  | 0.000 |
|  | CP+WLS-H | 50.34±5.42^**^ | 0.969 | 0.884 |  |  |  |  | 0.000 |
|  | CP+NPS2143 | 37.10±7.15^**^ | 0.975 | 0.923 |  |  |  |  | 0.000 |

Table 2-3. Blood urea nitrogen levels in cisplatin-induced AKI mice（*n*=6）**（Fig. 2D）**

| BUN | Group | Mean ± SD  （$\bar{x}\pm s$） | *Shapiro-Wilk* | | *levene test* | | *ANOVA* | | *LSD* |
| --- | --- | --- | --- | --- | --- | --- | --- | --- | --- |
|  |  |  | *W* | *P* | *F* | *P* | *F* | *P* | *P* |
|  | Control | 8.69±0.44 | 0.937 | 0.636 | 1.083 | 0.390 | 47.443 | 0.000 | - |
|  | CP | 14.48±1.22^##^ | 0.903 | 0.390 |  |  |  |  | 0.000 |
|  | CP+WLS-L | 15.05±1.47 | 0.838 | 0.127 |  |  |  |  | 0.351 |
|  | CP+WLS-M | 13.86±0.96 | 0.869 | 0.221 |  |  |  |  | 0.316 |
|  | CP+WLS-H | 14.12±0.78 | 0.896 | 0.353 |  |  |  |  | 0.555 |
|  | CP+NPS2143 | 8.72±1.11^**^ | 0.873 | 0.239 |  |  |  |  | 0.000 |

Table 2-4. KIM-1 mRNA expression in cisplatin-induced AKI mice（*n*=6）**（Fig. 2E）**

| KIM-1 | Group | Mean ± SD  （$\bar{x}\pm s$） | *Shapiro-Wilk* | | *levene test* | | *Welch* | | *Dunnett's T3* |
| --- | --- | --- | --- | --- | --- | --- | --- | --- | --- |
|  |  |  | *W* | *P* | *F* | *P* | *F* | *P* | *P* |
|  | Control | 0.01±0.00 | 0.901 | 0.377 | 5.822 | 0.001 | 165.769 | 0.000 | - |
|  | CP | 1.13±0.19^##^ | 0.852 | 0.163 |  |  |  |  | 0.000 |
|  | CP+WLS-L | 0.76±0.12^*^ | 0.836 | 0.122 |  |  |  |  | 0.043 |
|  | CP+WLS-M | 0.80±0.14 | 0.893 | 0.333 |  |  |  |  | 0.082 |
|  | CP+WLS-H | 0.85±0.11 | 0.916 | 0.476 |  |  |  |  | 0.143 |
|  | CP+NPS2143 | 0.03±0.01^**^ | 0.896 | 0.349 |  |  |  |  | 0.000 |

Table 2-5. Tubular injury scores in cisplatin-induced AKI mice（*n*=6）**(Fig. 2G）**

| Tubular injury score | Group | Mean ± SD  （$\bar{x}\pm s$） | *Shapiro-Wilk* | | *levene test* | | *ANOVA* | | *LSD* |
| --- | --- | --- | --- | --- | --- | --- | --- | --- | --- |
|  |  |  | *W* | *P* | *F* | *P* | *F* | *P* | *P* |
|  | Control | 0.17±0.41 | 0.692 | 0.603 | 0.439 | 0.818 | 42.814 | 0.000 | - |
|  | CP | 3.83±0.41 | 0.496 | 0.159 |  |  |  |  | 0.000 |
|  | CP+WLS-L | 3.33±0.52 | 0.640 | 0.758 |  |  |  |  | 0.087 |
|  | CP+WLS-M | 1.67±0.52 | 0.645 | 0.093 |  |  |  |  | 0.000 |
|  | CP+WLS-H | 2.00±0.63 | 0.827 | 0.068 |  |  |  |  | 0.000 |
|  | CP+NPS2143 | 1.83±0.41 | 0.493 | 0.117 |  |  |  |  | 0.000 |

**Statistical results for Figure 3**

Table 3-1. MCP-1 mRNA expression in cisplatin-induced AKI mice（*n*=6）**（Fig. 3A1）**

| MCP-1 | Group | Mean ± SD  （$\bar{x}\pm s$） | *Shapiro-Wilk* | | *levene test* | | *Welch* | | *Dunnett's T3* |
| --- | --- | --- | --- | --- | --- | --- | --- | --- | --- |
|  |  |  | *W* | *P* | *F* | *P* | *F* | *P* | *P* |
|  | Control | 0.10±0.04 | 0.958 | 0.808 | 4.603 | 0.003 | 52.814 | 0.000 | - |
|  | CP | 1.13±0.24^##^ | 0.870 | 0.187 |  |  |  |  | 0.000 |
|  | CP+WLS-L | 0.73±0.30 | 0.958 | 0.802 |  |  |  |  | 0.262 |
|  | CP+WLS-M | 0.60±0.26^*^ | 0.852 | 0.162 |  |  |  |  | 0.040 |
|  | CP+WLS-H | 0.61±0.10^*^ | 0.930 | 0.580 |  |  |  |  | 0.010 |
|  | CP+NPS2143 | 0.10±0.03^**^ | 0.905 | 0.404 |  |  |  |  | 0.000 |

Table 3-2. IL-1β mRNA expression in cisplatin-induced AKI mice（*n*=6）**（Fig. 3A2）**

| IL-1β | Group | Mean ± SD  *（*$\bar{x}\pm s$*）* | *Shapiro-Wilk* | | *levene test* | | *Welch* | | *Dunnett's T3* |
| --- | --- | --- | --- | --- | --- | --- | --- | --- | --- |
|  |  |  | *W* | *P* | *F* | *P* | *F* | *P* | *P* |
|  | Control | 0.51±0.09 | 0.958 | 0.808 | 5.029 | 0.002 | 13.53 | 0.000 | - |
|  | CP | 1.25±0.22^##^ | 0.870 | 0.187 |  |  |  |  | 0.002 |
|  | CP+WLS-L | 1.19±0.23 | 0.958 | 0.802 |  |  |  |  | 1.000 |
|  | CP+WLS-M | 0.79±0.10^*^ | 0.852 | 0.162 |  |  |  |  | 0.021 |
|  | CP+WLS-H | 0.97±0.05 | 0.930 | 0.580 |  |  |  |  | 0.196 |
|  | CP+NPS2143 | 0.25±0.08^**^ | 0.905 | 0.404 |  |  |  |  | 0.000 |

Table 3-3. TNF-α mRNA expression in cisplatin-induced AKI mice（*n*=6）**（Fig. 3A3）**

| TNF-α | Group | Mean ± SD  *（*$\bar{x}\pm s$*）* | *Shapiro-Wilk* | | *levene test* | | *ANOVA* | | *LSD* |
| --- | --- | --- | --- | --- | --- | --- | --- | --- | --- |
|  |  |  | *W* | *P* | *F* | *P* | *F* | *P* | *P* |
|  | Control | 0.18±0.048 | 0.861 | 0.193 | 1.131 | 0.375 | 101.761 | 0.000 | - |
|  | CP | 1.27±0.11^##^ | 0.810 | 0.121 |  |  |  |  | 0.000 |
|  | CP+WLS-L | 1.00±0.11^*^ | 0.950 | 0.718 |  |  |  |  | 0.017 |
|  | CP+WLS-M | 0.83±0.13^**^ | 0.933 | 0.614 |  |  |  |  | 0.006 |
|  | CP+WLS-H | 1.06±0.49^*^ | 0.950 | 0.896 |  |  |  |  | 0.012 |
|  | CP+NPS2143 | 0.41±0.09^**^ | 0.880 | 0.311 |  |  |  |  | 0.000 |

Table 3-4. F4/80 immunofluorescence in cisplatin-induced AKI mice（*n*=6）**（Fig. 3C）**

| F4/80 | Group | Mean ± SD  （$\bar{x}\pm s$） | *Shapiro-Wilk* | | *levene test* | | *ANOVA* | | *LSD* |
| --- | --- | --- | --- | --- | --- | --- | --- | --- | --- |
|  |  |  | *W* | *P* | *F* | *P* | *F* | *P* | *P* |
|  | Control | 0.12±0.04 | 0.951 | 0.746 | 1.796 | 0.180 | 329.638 | 0.000 | - |
|  | CP | 4.89±0.31^##^ | 0.871 | 0.231 |  |  |  |  | 0.000 |
|  | CP+WLS | 2.19±0.4^**^ | 0.883 | 0.282 |  |  |  |  | 0.000 |
|  | CP+NPS2143 | 1.36±0.21^**^ | 0.92 | 0.502 |  |  |  |  | 0.000 |

Table 3-5. GSH levels in cisplatin-induced AKI mice（*n*=6）**（Fig. 3D）**

| GSH | Group | Mean ± SD  （$\bar{x}\pm s$） | *Shapiro-Wilk* | | *levene test* | | *Welch* | | *Dunnett's T3* |
| --- | --- | --- | --- | --- | --- | --- | --- | --- | --- |
|  |  |  | *W* | *P* | *F* | *P* | *F* | *P* | *P* |
|  | Control | 24.01±3.60 | 0.838 | 0.124 | 6.613 | 0.000 | 30.696 | 0.000 | - |
|  | CP | 7.30±1.85^##^ | 0.957 | 0.786 |  |  |  |  | 0.000 |
|  | CP+WLS-L | 24.24±17.71 | 0.883 | 0.282 |  |  |  |  | 0.429 |
|  | CP+WLS-M | 31.81±19.63 | 0.902 | 0.384 |  |  |  |  | 0.212 |
|  | CP+WLS-H | 45.06±9.18^**^ | 0.950 | 0.739 |  |  |  |  | 0.001 |
|  | CP+NPS2143 | 16.54±9.40 | 0.940 | 0.658 |  |  |  |  | 0.412 |

Table 3-6. MDA levels in cisplatin-induced AKI mice（*n*=6）**（Fig. 3E）**

| MDA | Group | Mean ± SD  （$\bar{x}\pm s$） | *Shapiro-Wilk* | | *levene test* | | *ANOVA* | | *LSD* |
| --- | --- | --- | --- | --- | --- | --- | --- | --- | --- |
|  |  |  | *W* | *P* | *F* | *P* | *F* | *P* | *P* |
|  | Control | 3.43±0.63 | 0.809 | 0.096 | 0.871 | 0.515 | 30.696 | 0.000 | - |
|  | CP | 6.73±0.73^##^ | 0.962 | 0.818 |  |  |  |  | 0.000 |
|  | CP+WLS-L | 4.59±0.39^**^ | 0.953 | 0.761 |  |  |  |  | 0.000 |
|  | CP+WLS-M | 5.04±0.41^**^ | 0.875 | 0.289 |  |  |  |  | 0.000 |
|  | CP+WLS-H | 4.41±0.47^**^ | 0.992 | 0.986 |  |  |  |  | 0.000 |
|  | CP+NPS2143 | 3.02±0.78^**^ | 0.921 | 0.538 |  |  |  |  | 0.000 |

Table 3-7. SOD levels in cisplatin-induced AKI mice（*n*=6）**（Fig. 3F）**

| SOD | Group | Mean ± SD  （$\bar{x}\pm s$） | *Shapiro-Wilk* | | *levene test* | | *Welch* | | *Dunnett's T3* |
| --- | --- | --- | --- | --- | --- | --- | --- | --- | --- |
|  |  |  | *W* | *P* | *F* | *P* | *F* | *P* | *P* |
|  | Control | 35.77±3.84 | 0.809 | 0.096 | 2.931 | 0.032 | 25.674 | 0.000 | - |
|  | CP | 22.42±2.30^##^ | 0.962 | 0.818 |  |  |  |  | 0.001 |
|  | CP+WLS-L | 40.35±6.82^*^ | 0.953 | 0.761 |  |  |  |  | 0.024 |
|  | CP+WLS-M | 35.51±7.16 | 0.875 | 0.289 |  |  |  |  | 0.100 |
|  | CP+WLS-H | 34.34±2.98^**^ | 0.992 | 0.986 |  |  |  |  | 0.002 |
|  | CP+NPS2143 | 39.41±5.02^**^ | 0.921 | 0.538 |  |  |  |  | 0.006 |

Table 3-8. TUNEL fluorescence in cisplatin-induced AKI mice（*n*=6）**（Fig. 3H）**

| TUNEL | Group | Mean ± SD  （$\bar{x}\pm s$） | *Shapiro-Wilk* | | *levene test* | | *ANOVA* | | *LSD* |
| --- | --- | --- | --- | --- | --- | --- | --- | --- | --- |
|  |  |  | *W* | *P* | *F* | *P* | *F* | *P* | *P* |
|  | Control | 1.00±0.32 | 0.895 | 0.381 | 1.722 | 0.168 | 153.249 | 0.000 | - |
|  | CP | 10.95±1.14^###^ | 0.953 | 0.757 |  |  |  |  | 0.000 |
|  | CP+WLS | 2.96±0.41^***^ | 0.830 | 0.139 |  |  |  |  | 0.000 |
|  | CP+NPS2143 | 1.98±0.85^***^ | 0.846 | 0.182 |  |  |  |  | 0.000 |

Table 3-9. Bax/Bcl-2 protein expression in kidney tissues（*n*=3）**（Fig. 3J）**

| Bax/Bcl2 | Group | Mean ± SD  （$\bar{x}\pm s$） | *Shapiro-Wilk* | | *levene test* | | *Welch* | | *Dunnett's T3* |
| --- | --- | --- | --- | --- | --- | --- | --- | --- | --- |
|  |  |  | *W* | *P* | *F* | *P* | *F* | *P* | *P* |
|  | Control | 0.28±0.14 | 0.789 | 0.088 | 3.245 | 0.044 | 36.556 | 0.000 | - |
|  | CP | 1.50±0.10^##^ | 0.928 | 0.482 |  |  |  |  | 0.003 |
|  | CP+WLS-L | 1.20±0.26 | 0.837 | 0.208 |  |  |  |  | 0.651 |
|  | CP+WLS-M | 0.77±0.18^*^ | 0.923 | 0.462 |  |  |  |  | 0.045 |
|  | CP+WLS-H | 0.91±0.07^*^ | 0.912 | 0.425 |  |  |  |  | 0.015 |
|  | CP+NPS2143 | 0.48±0.06^**^ | 0.956 | 0.597 |  |  |  |  | 0.003 |

Table 3-10. Cleaved caspase-3/caspase-3 protein expression in kidney tissues（*n*=3）**（Fig. 3K）**

| Cleaved Caspase 3/ Caspase 3 | Group | Mean ± SD  （$\bar{x}\pm s$） | *Shapiro-Wilk* | | *levene test* | | *ANOVA* | | *LSD* |
| --- | --- | --- | --- | --- | --- | --- | --- | --- | --- |
|  |  |  | *W* | *P* | *F* | *P* | *F* | *P* | *P* |
|  | Control | 0.28±0.14 | 0.966 | 0.644 | 2.624 | 0.079 | 3.153 | 0.048 | - |
|  | CP | 1.50±0.10^##^ | 0.999 | 0.948 |  |  |  |  | 0.019 |
|  | CP+WLS-L | 1.20±0.26 | 0.995 | 0.871 |  |  |  |  | 0.020 |
|  | CP+WLS-M | 0.77±0.18^*^ | 0.886 | 0.343 |  |  |  |  | 0.007 |
|  | CP+WLS-H | 0.91±0.07^*^ | 0.782 | 0.073 |  |  |  |  | 0.005 |
|  | CP+NPS2143 | 0.48±0.06^**^ | 0.848 | 0.236 |  |  |  |  | 0.015 |

**Statistical results for Figure 4**

Table 4-1. mRTEC cell viability at different cisplatin concentrations（*n*=6）**(Fig. 4A）**

| CP | Group | Mean ± SD  （$\bar{x}\pm s$） | *Shapiro-Wilk* | | *levene test* | | *Welch* | | *Dunnett's T3* |
| --- | --- | --- | --- | --- | --- | --- | --- | --- | --- |
|  |  |  | *W* | *P* | *F* | *P* | *F* | *P* | *P* |
|  | Control | 100.00±2.21 | 0.914 | 0.254 | 5.561 | 0.000 | 3262.888 | 0.000 | - |
|  | CP-2.5µM | 92.93±2.92^##^ | 0.947 | 0.818 |  |  |  |  | 0.000 |
|  | CP-5µM | 77.55±1.35^##^ | 0.934 | 0.761 |  |  |  |  | 0.000 |
|  | CP-10µM | 37.09±1.62^#^^#^ | 0.910 | 0.289 |  |  |  |  | 0.000 |
|  | CP-20µM | 18.27±0.32^##^ | 0.870 | 0.228 |  |  |  |  | 0.000 |
|  | CP-40µM | 18.51±0.47^##^ | 0.957 | 0.796 |  |  |  |  | 0.000 |
|  | CP-80µM | 6.98±1.09^##^ | 0.952 | 0.752 |  |  |  |  | 0.000 |

Table 4-2. mRTEC cell viability after WLS-containing serum treatment（*n*=6）**(Fig. 4B）**

| WLS | Group | Mean ± SD  （$\bar{x}\pm s$） | *Shapiro-Wilk* | | *levene test* | | *ANOVA* | | *LSD* |
| --- | --- | --- | --- | --- | --- | --- | --- | --- | --- |
|  |  |  | *W* | *P* | *F* | *P* | *F* | *P* | *P* |
|  | Control | 100.00±5.96 | 0.815 | 0.079 | 0.821 | 0.524 | 2.538 | 0.065 | - |
|  | 2.5%WLS | 99.70±5.31 | 0.864 | 0.204 |  |  |  |  | 0.917 |
|  | 5%WLS | 98.54±5.28 | 0.884 | 0.288 |  |  |  |  | 0.620 |
|  | 10%WLS | 98.18±4.96 | 0.903 | 0.393 |  |  |  |  | 0.538 |
|  | 15%WLS | 91.98±3.26^#^ | 0.829 | 0.106 |  |  |  |  | 0.011 |

Table 4-3. ROS levels in cisplatin-treated mRTECs（*n=*6）**(Fig. 4C）**

| ROS | Group | Mean ± SD  （$\bar{x}\pm s$） | *Shapiro-Wilk* | | *levene test* | | *ANOVA* | | *LSD* |
| --- | --- | --- | --- | --- | --- | --- | --- | --- | --- |
|  |  |  | *W* | *P* | *F* | *P* | *F* | *P* | *P* |
|  | Control | 62.17±2.75 | 0.824 | 0.174 | 0.556 | 0.659 | 23.891 | 0.000 | - |
|  | CP | 84.00±4.01^##^ | 0.993 | 0.835 |  |  |  |  | 0.000 |
|  | CP+5%WLS | 76.40±3.77^*^ | 0.987 | 0.780 |  |  |  |  | 0.020 |
|  | CP+10%WLS | 76.07±1.90^*^ | 0.996 | 0.884 |  |  |  |  | 0.017 |

Table 4-4. Apoptosis rate in cisplatin-treated mRTECs（*n=*6）**(Fig. 4D）**

| TUNEL | Group | Mean ± SD  （$\bar{x}\pm s$） | *Shapiro-Wilk* | | *levene test* | | *ANOVA* | | *LSD* |
| --- | --- | --- | --- | --- | --- | --- | --- | --- | --- |
|  |  |  | *W* | *P* | *F* | *P* | *F* | *P* | *P* |
|  | Control | 12.28±2.56 | 0.967 | 0.654 | 0.769 | 0.543 | 61.022 | 0.000 | - |
|  | CP | 47.50±3.16^##^ | 0.873 | 0.303 |  |  |  |  | 0.000 |
|  | CP+5%WLS | 40.77±2.80^*^ | 0.998 | 0.921 |  |  |  |  | 0.042 |
|  | CP+10%WLS | 37.33±4.70^**^ | 0.937 | 0.514 |  |  |  |  | 0.006 |

Table 4-5. Bax/Bcl-2 protein expression in cisplatin-treated mRTECs（*n=*3）**(Fig. 4H）**

| Bax/Bcl2 | Group | Mean ± SD  （$\bar{x}\pm s$） | *Shapiro-Wilk* | | *levene test* | | *ANOVA* | | *LSD* |
| --- | --- | --- | --- | --- | --- | --- | --- | --- | --- |
|  |  |  | *W* | *P* | *F* | *P* | *F* | *P* | *P* |
|  | Control | 0.8±0.09 | 0.998 | 0.906 | 1.676 | 0.248 | 14.037 | 0.001 | - |
|  | CP | 1.27±0.16^##^ | 0.971 | 0.673 |  |  |  |  | 0.001 |
|  | CP+5%WLS | 0.91±0.05^**^ | 0.854 | 0.252 |  |  |  |  | 0.003 |
|  | CP+10%WLS | 0.8±0.06^**^ | 0.753 | 0.056 |  |  |  |  | 0.001 |

Table 4-6. Cleaved caspase-3/caspase-3 protein expression in cisplatin-treated mRTECs（*n=*3）**(Fig. 4I）**

| Cleaved Caspase 3/Caspase 3 | Group | Mean ± SD  （$\bar{x}\pm s$） | *Shapiro-Wilk* | | *levene test* | | *ANOVA* | | *LSD* |
| --- | --- | --- | --- | --- | --- | --- | --- | --- | --- |
|  |  |  | *W* | *P* | *F* | *P* | *F* | *P* | *P* |
|  | Control | 0.19±0.02 | 0.986 | 0.772 | 3.606 | 0.065 | 8.132 | 0.008 | - |
|  | CP | 0.52±0.09^##^ | 0.802 | 0.119 |  |  |  |  | 0.001 |
|  | CP+5%WLS | 0.32±0.14^*^ | 0.967 | 0.649 |  |  |  |  | 0.019 |
|  | CP+10%WLS | 0.29±0.04^*^ | 0.835 | 0.201 |  |  |  |  | 0.011 |

**Statistical results for Figure 5**

Table 5-1A. GPCR mRNA expression in AKI mice (normally distributed)（*n*=3）**(Fig. 5A1）**

| Gene | Group | Mean ± SD  （$\bar{x}\pm s$） | *Shapiro-Wilk* | | *levene test* | | *ANOVA* | | *LSD* |
| --- | --- | --- | --- | --- | --- | --- | --- | --- | --- |
|  |  |  | *W* | *P* | *F* | *P* | *F* | *P* | *P* |
| CaSR | Control | 3.41±0.11 | 0.998 | 0.906 | 0.796 | 0.494 | 122.019 | 0.000 | - |
|  | AKI | 0.59±0.23^###^ | 1.000 | 0.987 |  |  |  |  | 0.000 |
|  | WLS | 2.15±0.28^***^ | 0.992 | 0.824 |  |  |  |  | 0.000 |
| HTR2A | Control | 1.96±0.24 | 0.919 | 0.448 | 0.106 | 0.901 | 16.282 | 0.004 | - |
|  | AKI | 0.93±0.20^###^ | 0.785 | 0.079 |  |  |  |  | 0.002 |
|  | WLS | 1.84±0.26^***^ | 0.969 | 0.659 |  |  |  |  | 0.003 |
| CXCR4 | Control | 3.42±0.49 | 0.955 | 0.594 | 0.248 | 0.788 | 5.122 | 0.050 | - |
|  | AKI | 2.14±0.69 | 0.966 | 0.645 |  |  |  |  | 0.035 |
|  | WLS | 2.08±0.54 | 1.000 | 0.995 |  |  |  |  | 0.918 |
| P2Y2R | Control | 2.3±0.41 | 0.992 | 0.825 | 0.321 | 0.737 | 9.176 | 0.015 | - |
|  | AKI | 1.27±0.25 | 0.967 | 0.652 |  |  |  |  | 0.011 |
|  | WLS | 1.22±0.37 | 0.986 | 0.774 |  |  |  |  | 0.874 |
| EATR | Control | 1.11±0.12 | 0.964 | 0.634 | 0.767 | 0.505 | 0.254 | 0.784 | - |
|  | AKI | 1.04±0.09 | 0.813 | 0.147 |  |  |  |  | 0.550 |
|  | WLS | 1.1±0.17 | 0.946 | 0.551 |  |  |  |  | 0.571 |
| P2X4R | Control | 1.48±0.16 | 0.811 | 0.142 | 1.209 | 0.362 | 20.693 | 0.002 | - |
|  | AKI | 1.05±0.09 | 0.981 | 0.732 |  |  |  |  | 0.007 |
|  | WLS | 0.82±0.12 | 0.909 | 0.415 |  |  |  |  | 0.059 |
| S1PR2 | Control | 0.68±0.03 | 0.999 | 0.941 | 1.378 | 0.322 | 20.072 | 0.002 | - |
|  | AKI | 10.94±0.12 | 1.000 | 0.976 |  |  |  |  | 0.007 |
|  | WLS | 1.09±0.06 | 0.958 | 0.608 |  |  |  |  | 0.069 |
| CNR1 | Control | 1.2±0.61 | 0.997 | 0.896 | 2.838 | 0.136 | 0.657 | 0.552 | - |
|  | AKI | 1.01±0.14 | 0.999 | 0.934 |  |  |  |  | 0.545 |
|  | WLS | 0.86±0.11 | 0.846 | 0.230 |  |  |  |  | 0.634 |

Table 5-1B. GPCR mRNA expression in AKI mice (non-normally distributed)（*n*=3）**(Fig. 5A2）**

| Gene | Group | Mean ± SD  （$\bar{x}\pm s$） | *Shapiro-Wilk* | | *Kruskal-Wallis* |
| --- | --- | --- | --- | --- | --- |
|  |  |  | *W* | *P* | *P* |
| HTR4 | Control | 2.6±0.73 | 0.780 | 0.067 | 0.027 |
|  | CP | 0.77±0.29^#^ | 0.756 | 0.012 |  |
|  | WLS | 1.35±0.43 | 0.988 | 0.791 |  |
| SUCNR1 | Control | 3.92±1.59 | 0.939 | 0.525 | 0.113 |
|  | AKI | 1.21±0.93 | 0.858 | 0.262 |  |
|  | WLS | 1.41±0.27 | 0.763 | 0.028 |  |
| S1PR3 | Control | 1.48±0.37 | 0.757 | 0.016 | 0.061 |
|  | AKI | 0.84±0.11 | 0.902 | 0.390 |  |
|  | WLS | 0.78±0.21 | 0.851 | 0.243 |  |

Table 5-2. CaSR protein expression in kidney tissues (two groups)（*n*=5） **(Fig. 5D）**

| CaSR | Group | Mean ± SD  （$\bar{x}\pm s$） | *Shapiro-Wilk* | | *levene test* | | *t* |
| --- | --- | --- | --- | --- | --- | --- | --- |
|  |  |  | *W* | *P* | *F* | *P* | *P* |
|  | Control | 0.64±0.07 | 0.929 | 0.573 | 1.330 | 0.279 | 0.000 |
|  | CP | 1.07±0.05^##^ | 0.997 | 0.998 |  |  |  |

Table 5-3. CaSR protein expression in kidney tissues (multi-group)（*n*=3）**(Fig. 5F1）**

| CaSR/β-Actin | Group | Mean ± SD  （$\bar{x}\pm s$） | *Shapiro-Wilk* | | *levene test* | | *ANOVA* | | *LSD* |
| --- | --- | --- | --- | --- | --- | --- | --- | --- | --- |
|  |  |  | *W* | *P* | *F* | *P* | *F* | *P* | *P* |
|  | Control | 0.38±0.21 | 0.937 | 0.639 | 2.455 | 0.073 | 3.829 | 0.015 | - |
|  | CP | 0.82±0.30^#^ | 0.972 | 0.852 |  |  |  |  | 0.014 |
|  | CP+WLS-L | 0.81±0.41 | 0.908 | 0.472 |  |  |  |  | 0.937 |
|  | CP+WLS-M | 0.47±0.11^*^ | 0.795 | 0.094 |  |  |  |  | 0.045 |
|  | CP+WLS-H | 0.48±0.04 | 0.934 | 0.615 |  |  |  |  | 0.051 |
|  | CP+NPS2143 | 0.28±0.01^**^ | 0.846 | 0.214 |  |  |  |  | 0.004 |

Table 5-4. CaMKKβ protein expression in kidney tissues（*n*=3）**(Fig. 5F2）**

| CaMKKβ/β-Actin | Group | Mean ± SD  （$\bar{x}\pm s$） | *Shapiro-Wilk* | | *levene test* | | *ANOVA* | | *LSD* |
| --- | --- | --- | --- | --- | --- | --- | --- | --- | --- |
|  |  |  | *W* | *P* | *F* | *P* | *F* | *P* | *P* |
|  | Control | 0.25±0.01 | 0.911 | 0.420 | 2.426 | 0.097 | 23.024 | 0.000 | - |
|  | CP | 0.81±0.05^#^ | 0.812 | 0.144 |  |  |  |  | 0.000 |
|  | CP+WLS-L | 0.76±0.11 | 0.986 | 0.776 |  |  |  |  | 0.488 |
|  | CP+WLS-M | 0.43±0.09^*^ | 0.919 | 0.449 |  |  |  |  | 0.000 |
|  | CP+WLS-H | 0.55±0.14 | 0.835 | 0.200 |  |  |  |  | 0.004 |
|  | CP+NPS2143 | 0.23±0.02^**^ | 0.964 | 0.635 |  |  |  |  | 0.000 |

Table 5-5. p-AMPK protein expression in kidney tissues（*n*=3）**(Fig. 5H3）**

| p-AMPK/β-Actin | Group | Mean ± SD  （$\bar{x}\pm s$） | *Shapiro-Wilk* | | *levene test* | | *ANOVA* | | *LSD* |
| --- | --- | --- | --- | --- | --- | --- | --- | --- | --- |
|  |  |  | *W* | *P* | *F* | *P* | *F* | *P* | *P* |
|  | Control | 0.35±0.12 | 0.998 | 0.914 | 0.711 | 0.627 | 4.525 | 0.015 | - |
|  | CP | 0.61±0.11# | 0.909 | 0.413 |  |  |  |  | 0.014 |
|  | CP+WLS-L | 0.34±0.15 | 0.808 | 0.133 |  |  |  |  | 0.013 |
|  | CP+WLS-M | 0.33±0.14* | 0.992 | 0.829 |  |  |  |  | 0.009 |
|  | CP+WLS-H | 0.28±0.08 | 0.801 | 0.117 |  |  |  |  | 0.003 |
|  | CP+NPS2143 | 0.20±0.06** | 0.981 | 0.739 |  |  |  |  | 0.001 |

Table 5-6. CaSR immunofluorescence intensity in kidney tissues（*n*=6）**(Fig. 5H）**

| CaSR | Group | Mean ± SD  （$\bar{x}\pm s$） | *Shapiro-Wilk* | | *levene test* | | *Welch* | | *Dunnett's T3* |
| --- | --- | --- | --- | --- | --- | --- | --- | --- | --- |
|  |  |  | *W* | *P* | *F* | *P* | *F* | *P* | *P* |
|  | Control | 1.00±0.24 | 0.876 | 0.252 | 19.978 | 0.000 | 59.808 | 0.000 | - |
|  | CP | 10.35±2.67^##^ | 0.930 | 0.582 |  |  |  |  | 0.002 |
|  | CP+WLS-M | 3.21±0.63^**^ | 0.933 | 0.604 |  |  |  |  | 0.005 |
|  | CP+NPS2143 | 1.28±0.31^**^ | 0.938 | 0.645 |  |  |  |  | 0.002 |

Table 5-7. Pearson‘s correlation coefficient for CaSR/AQP1 colocalization（*n*=6）**(Fig. 5I）**

| Pearson's correlation coefficients | Group | Mean ± SD  （$\bar{x}\pm s$） | *Shapiro-Wilk* | | *levene test* | | *ANOVA* | | *LSD* |
| --- | --- | --- | --- | --- | --- | --- | --- | --- | --- |
|  |  |  | *W* | *P* | *F* | *P* | *F* | *P* | *P* |
|  | Control | -0.26±0.04 | 0.913 | 0.454 | 1.029 | 0.401 | 34.468 | 0.000 | - |
|  | CP | 0±0.06^##^ | 0.884 | 0.287 |  |  |  |  | 0.000 |
|  | CP+WLS-M | -0.08±0.04^**^ | 0.802 | 0.061 |  |  |  |  | 0.007 |
|  | CP+NPS2143 | -0.19±0.06^**^ | 0.850 | 0.156 |  |  |  |  | 0.000 |

Table 5-8. Manders’ M1 coefficient for CaSR/AQP1 colocalization（*n*=6）**(Fig. 5J）**

| M1 | Group | Mean ± SD  （$\bar{x}\pm s$） | *Shapiro-Wilk* | | *levene test* | | *ANOVA* | | *LSD* |
| --- | --- | --- | --- | --- | --- | --- | --- | --- | --- |
|  |  |  | *W* | *P* | *F* | *P* | *F* | *P* | *P* |
|  | Control | 0.29±0.13 | 0.863 | 0.201 | 1.075 | 0.382 | 10.295 | 0.000 | - |
|  | CP | 0.28±0.06 | 0.919 | 0.499 |  |  |  |  | 0.915 |
|  | CP+WLS-M | 0.59±0.1^##^ | 0.892 | 0.328 |  |  |  |  | 0.000 |
|  | CP+NPS2143 | 0.24±0.17 | 0.854 | 0.169 |  |  |  |  | 0.601 |

Table 5-9. Manders‘ M2 coefficient for CaSR/AQP1 colocalization（*n*=6）**(Fig. 5J）**

| M2 | Group | Mean ± SD  （$\bar{x}\pm s$） | *Shapiro-Wilk* | | *Kruskal-Wallis* | *P* |
| --- | --- | --- | --- | --- | --- | --- |
|  |  |  | *W* | *P* | *P* | *P* |
|  | Control | 0.03 ± 0.02 | 0.951 | 0.748 | 0.000 | - |
|  | CP | 0.75 ± 0.13^##^ | 0.854 | 0.169 |  | 0.004 |
|  | CP+WLS-M | 0.24 ± 0.06 | 0.925 | 0.540 |  | 0.084 |
|  | CP+NPS2143 | 0.02 ± 0.02^**^ | 0.757 | 0.023 |  | 0.001 |

Table 5-10. CaSR immunohistochemical staining (IOD) in kidney tissues（*n*=6）**(Fig. 5L）**

| CaSR | Group | Mean ± SD  （$\bar{x}\pm s$） | *Shapiro-Wilk* | | *levene test* | | *ANOVA* | | *LSD* |
| --- | --- | --- | --- | --- | --- | --- | --- | --- | --- |
|  |  |  | *W* | *P* | *F* | *P* | *F* | *P* | *P* |
|  | Control | 0.10±0.01 | 0.915 | 0.469 | 2.165 | 0.085 | 30.696 | 0.000 | - |
|  | CP | 0.16±0.02^##^ | 0.834 | 0.115 |  |  |  |  | 0.000 |
|  | CP+WLS | 0.09±0.00^**^ | 0.910 | 0.436 |  |  |  |  | 0.000 |
|  | CP+NPS2143 | 0.10±0.01^**^ | 0.976 | 0.930 |  |  |  |  | 0.000 |

Table 5-11. CaMKKβ immunohistochemical staining (IOD) in kidney tissues（*n*=6）**(Fig. 5M）**

| CaMKKβ | Group | Mean ± SD  （$\bar{x}\pm s$） | *Shapiro-Wilk* | | *levene test* | | *ANOVA* | | *LSD* |
| --- | --- | --- | --- | --- | --- | --- | --- | --- | --- |
|  |  |  | *W* | *P* | *F* | *P* | *F* | *P* | *P* |
|  | Control | 0.09±0.01 | 0.865 | 0.208 | 1.812 | 0.141 | 80.883 | 0.000 | - |
|  | CP | 0.13±0.01^##^ | 0.807 | 0.068 |  |  |  |  | 0.000 |
|  | CP+WLS | 0.08±0.01^**^ | 0.871 | 0.232 |  |  |  |  | 0.000 |
|  | CP+NPS2143 | 0.10±0.00^**^ | 0.856 | 0.177 |  |  |  |  | 0.000 |

**Statistical results for Figure 6**

Table 6-1. CaSR protein expression in cisplatin-treated mRTECs（*n*=3）**(Fig. 6B1）**

| CaSR/β-actin | Group | Mean ± SD  （$\bar{x}\pm s$） | *Shapiro-Wilk* | | *levene test* | | *t* |
| --- | --- | --- | --- | --- | --- | --- | --- |
|  |  |  | *W* | *P* | *F* | *P* | *P* |
|  | Control | 1.17±0.40 | 0.908 | 0.410 | 1.111 | 0.351 | 0.037 |
|  | CP | 2.01±0.26^#^ | 0.945 | 0.549 |  |  |  |

Table 6-2. Bax/Bcl-2 protein expression in cisplatin-treated mRTECs（*n*=3）**(Fig. 6B2）**

| Bax/Bcl2 | Group | Mean ± SD  （$\bar{x}\pm s$） | *Shapiro-Wilk* | | *levene test* | | *t* |
| --- | --- | --- | --- | --- | --- | --- | --- |
|  |  |  | *W* | *P* | *F* | *P* | *P* |
|  | Control | 1.18±0.34 | 0.994 | 0.849 | 4.682 | 0.096 | 0.006 |
|  | CP | 2.21±0.36^##^ | 0.772 | 0.051 |  |  |  |

Table 6-3. Intracellular Ca²⁺ levels in cisplatin-treated mRTECs（*n*=3）**(Fig. 6C）**

| Ca^2+^ | Group | Mean ± SD  （$\bar{x}\pm s$） | *Shapiro-Wilk* | | *levene test* | | *ANOVA* | | *LSD* |
| --- | --- | --- | --- | --- | --- | --- | --- | --- | --- |
|  |  |  | *W* | *P* | *F* | *P* | *F* | *P* | *P* |
|  | Control | 2.51±0.15 | 0.875 | 0.310 | 3.034 | 0.093 | 46.017 | 0.000 | - |
|  | CP | 3.54±0.16^##^ | 0.873 | 0.305 |  |  |  |  | 0.000 |
|  | CP+5%WLS | 2.67±0.04^**^ | 0.782 | 0.073 |  |  |  |  | 0.000 |
|  | CP+10%WLS | 2.69±0.08^**^ | 0.992 | 0.832 |  |  |  |  | 0.000 |

Table 6-4. Cinacalcet cytotoxicity in mRTECs（*n*=6）**(Fig. 6D）**

| Cinacalcet | Group | Mean ± SD  （$\bar{x}\pm s$） | *Shapiro-Wilk* | | *levene test* | | *ANOVA* | | *LSD* |
| --- | --- | --- | --- | --- | --- | --- | --- | --- | --- |
|  |  |  | *W* | *P* | *F* | *P* | *F* | *P* | *P* |
|  | Control | 100.00±3.41 | 0.983 | 0.964 | 0.870 | 0.513 | 2.112 | 0.091 | - |
|  | 0.625 | 98.54±2 | 0.937 | 0.636 |  |  |  |  | 0.546 |
|  | 1.25 | 100.66±5.2 | 0.927 | 0.560 |  |  |  |  | 0.382 |
|  | 2.5 | 98.58±3.98 | 0.972 | 0.902 |  |  |  |  | 0.987 |
|  | 5 | 96.11±4.9 | 0.998 | 1.000 |  |  |  |  | 0.315 |
|  | 10 | 94.17±4.44 | 0.921 | 0.510 |  |  |  |  | 0.076 |

Table 6-5. NPS2143 cytotoxicity in mRTECs（*n*=6）**(Fig. 6E）**

| NPS2143 | Group | Mean ± SD  （$\bar{x}\pm s$） | *Shapiro-Wilk* | | *levene test* | | *ANOVA* | | *LSD* |
| --- | --- | --- | --- | --- | --- | --- | --- | --- | --- |
|  |  |  | *W* | *P* | *F* | *P* | *F* | *P* | *P* |
|  | Control | 100.00±2.41 | 0.872 | 0.235 | 1.329 | 0.279 | 1296.517 | 0.000 | - |
|  | 0.625 | 102.73±3.73 | 0.925 | 0.543 |  |  |  |  | 0.094 |
|  | 1.25 | 101.85±2.29 | 0.995 | 0.997 |  |  |  |  | 0.582 |
|  | 2.5 | 103.15±2.32 | 0.952 | 0.753 |  |  |  |  | 0.792 |
|  | 5 | 98±3.72 | 0.890 | 0.318 |  |  |  |  | 0.055 |
|  | 10 | 3.02±0.74^##^ | 0.952 | 0.758 |  |  |  |  | 0.000 |

Table 6-6A. Bax/Bcl-2 protein expression in mRTECs（*n*=3）**(Fig. 6G）**

| Bax/Bcl2 | Group | Mean ± SD  （$\bar{x}\pm s$） | *Shapiro-Wilk* | | *levene test* | | *ANOVA* | | *LSD* |
| --- | --- | --- | --- | --- | --- | --- | --- | --- | --- |
|  |  |  | *W* | *P* | *F* | *P* | *F* | *P* | *P* |
|  | Control | 0.97±0.22 | 0.937 | 0.514 | 2.350 | 0.105 | 5.574 | 0.007 | - |
|  | CP | 1.32±0.15^#^ | 0.902 | 0.392 |  |  |  |  | 0.014 |
|  | CP+5%WLS | 0.94±0.06^**^ | 0.844 | 0.226 |  |  |  |  | 0.009 |
|  | CP+NPS2143 | 0.96±0.02^*^ | 0.999 | 0.930 |  |  |  |  | 0.011 |
|  | CP+Cinacalcet | 1.38±0.2 | 0.985 | 0.763 |  |  |  |  | 0.632 |
|  | CP+Cinacalcet+5%WLS | 1.26±0.15 | 0.933 | 0.499 |  |  |  |  | 0.620 |

Table 6-6B. Cleaved caspase-3/caspase-3 protein expression in mRTECs（*n*=3）**(Fig. 6H）**

| Cleaved Caspase 3/Caspase 3 | Group | Mean ± SD  （$\bar{x}\pm s$） | *Shapiro-Wilk* | | *levene test* | | *ANOVA* | | *LSD* |
| --- | --- | --- | --- | --- | --- | --- | --- | --- | --- |
|  |  |  | *W* | *P* | *F* | *P* | *F* | *P* | *P* |
|  | Control | 0.22±0.05 | 0.788 | 0.087 | 0.499 | 0.773 | 6.090 | 0.005 | - |
|  | CP | 0.39±0.05^##^ | 0.999 | 0.931 |  |  |  |  | 0.001 |
|  | CP+5%WLS | 0.27±0.04^*^ | 0.884 | 0.338 |  |  |  |  | 0.012 |
|  | CP+NPS2143 | 0.26±0.06^**^ | 0.963 | 0.628 |  |  |  |  | 0.008 |
|  | CP+Cinacalcet | 0.39±0.06 | 0.999 | 0.948 |  |  |  |  | 0.967 |
|  | CP+Cinacalcet+5%WLS | 0.3±0.02 | 0.908 | 0.413 |  |  |  |  | 0.055 |

**Statistical results for Figure 7**

Table 7-1. CaSR protein expression in mRTECs (multi-group)（*n*=3）**(Fig. 7B）**

| CaSR | Group | Mean ± SD  （$\bar{x}\pm s$） | *Shapiro-Wilk* | | *levene test* | | *ANOVA* | | *LSD* |
| --- | --- | --- | --- | --- | --- | --- | --- | --- | --- |
|  |  |  | *W* | *P* | *F* | *P* | *F* | *P* | *P* |
|  | Control | 1.22±0.03 | 0.813 | 0.145 | 2.102 | 0.135 | 9.637 | 0.001 | - |
|  | CP | 1.82±0.17^##^ | 0.985 | 0.764 |  |  |  |  | 0.001 |
|  | CP+5%WLS | 1.46±0.21^*^ | 0.803 | 0.122 |  |  |  |  | 0.019 |
|  | CP+NPS2143 | 1.22±0.16^**^ | 0.987 | 0.778 |  |  |  |  | 0.001 |
|  | CP+Cinacalcet | 1.85±0.22 | 0.896 | 0.373 |  |  |  |  | 0.816 |
|  | CP+Cinacalcet+5%WLS | 1.76±0.09 | 0.987 | 0.782 |  |  |  |  | 0.641 |

Table 7-2. CaMKKβ protein expression in mRTECs (multi-group)（*n*=3）**(Fig. 7C）**

| CaMKKβ | Group | Mean ± SD  （$\bar{x}\pm s$） | *Shapiro-Wilk* | | *levene test* | | *ANOVA* | | *LSD* |
| --- | --- | --- | --- | --- | --- | --- | --- | --- | --- |
|  |  |  | *W* | *P* | *F* | *P* | *F* | *P* | *P* |
|  | Control | 1.13±0.13 | 0.971 | 0.673 | 0.938 | 0.491 | 11.439 | 0.000 | - |
|  | CP | 1.52±0.07^##^ | 0.909 | 0.416 |  |  |  |  | 0.001 |
|  | CP+5%WLS | 1.28±0.1^*^ | 0.913 | 0.428 |  |  |  |  | 0.025 |
|  | CP+NPS2143 | 1.1±0.08^**^ | 0.977 | 0.711 |  |  |  |  | 0.001 |
|  | CP+Cinacalcet | 1.56±0.19 | 0.998 | 0.921 |  |  |  |  | 0.684 |
|  | CP+Cinacalcet+5%WLS | 1.6±0.07 | 0.950 | 0.568 |  |  |  |  | 0.426 |

Table 7-3. p-AMPK protein expression in mRTECs (multi-group)（*n*=3）**(Fig. 7D）**

| p-AMPK | Group | Mean ± SD  （$\bar{x}\pm s$） | *Shapiro-Wilk* | | *levene test* | | *ANOVA* | | *LSD* |
| --- | --- | --- | --- | --- | --- | --- | --- | --- | --- |
|  |  |  | *W* | *P* | *F* | *P* | *F* | *P* | *P* |
|  | Control | 0.62±0.17 | 0.833 | 0.177 | 0.325 | 0.891 | 3.193 | 0.031 | - |
|  | CP | 1.1±0.16^##^ | 0.984 | 0.926 |  |  |  |  | .004 |
|  | CP+5%WLS | 0.93±0.19 | 0.986 | 0.937 |  |  |  |  | .187 |
|  | CP+NPS2143 | 0.91±0.23 | 0.978 | 0.892 |  |  |  |  | .209 |
|  | CP+Cinacalcet | 1.13±0.18 | 0.979 | 0.897 |  |  |  |  | .922 |
|  | CP+Cinacalcet+5%WLS | 0.97±0.15 | 0.950 | 0.718 |  |  |  |  | .120 |

Statistical note: All data are presented as mean ± SD. Normality was assessed using the Shapiro-Wilk test. Homogeneity of variances was assessed using Levene‘s test. For multiple comparisons: (1) When data were normally distributed with equal variances (Levene’s test *P* > 0.05), one-way ANOVA followed by LSD post-hoc test was used. (2) When data were normally distributed with unequal variances (Levene‘s test *P* < 0.05), Welch‘s ANOVA followed by Dunnett’s T3 post-hoc test was used. (3) When data were not normally distributed (Shapiro-Wilk test *P* < 0.05), the Kruskal-Wallis test followed by Dunn-Bonferroni post-hoc test was used. ^#^*P* < 0.05, ^##^*P* < 0.01 vs. control group; ^*^*P* < 0.05, ^**^*P* < 0.01 vs. model group.
